# A Framework for Global Multicategory and Multiscalar Drought Characterization Accounting for Snow Processes

**DOI:** 10.1029/2019WR025529

**Published:** 2019-11-19

**Authors:** Baoqing Zhang, Youlong Xia, Laurie S. Huning, Jiahua Wei, Guangqian Wang, Amir AghaKouchak

**Affiliations:** ^1^ Key Laboratory of Western China's Environmental Systems (Ministry of Education) College of Earth and Environmental Sciences Lanzhou University Lanzhou China; ^2^ State Key Laboratory of Hydroscience and Engineering Department of Hydraulic Engineering Tsinghua University Beijing China; ^3^ I. M. Systems Group at Environmental Modeling Center National Centers for Environmental Prediction College Park MD USA; ^4^ Department of Civil and Environmental Engineering University of California Irvine CA USA; ^5^ Department of Earth System Science University of California Irvine CA USA

## Abstract

Drought indices do not always provide the most relevant information for water resources management as most of them neglect the role of snow in the land surface water balance. In this study, a physically based drought index, the Standardized Moisture Anomaly Index (SZI), was modified and improved by incorporating the effects of snow dynamics for drought characterization at multiple time scales. The new version of the SZI, called SZI_snow_, includes snow in both the water supply and demand in drought characterization by using the water‐energy budgets from the Global Land Data Assimilation Systems product. We compared and evaluated the performance of SZI_snow_ and SZI in drought identification globally across various time scales using observed multicategory drought evidences from several sources. Results show that the SZI_snow_ agrees better with the observed changes in hydrological and agricultural droughts than the SZI, particularly over basins with high snow accumulation. Furthermore, the SZI_snow_ is more consistent with the residual water‐energy ratio than the SZI over snow‐influenced regions. Overall, the SZI_snow_ can be either a complement or an improvement over the SZI for identifying, monitoring, and characterizing hydrological and agricultural droughts at various scales (e.g., 1–48 months) over high‐latitude and high‐elevation regions that receive snow.

## Key Points

- A global multi‐scalar drought index (SZI_snow_) was developed to improve the drought characterization by incorporating snow processes
- The SZI_snow_ generally performs better than the SZI for monitoring multi‐category droughts by considering snow dynamics
- The SZI_snow_ incorporates water‐energy budgets to address the influence of snow on both the water supply and demand in drought assessment

## 1 Introduction

Determining the occurrence and evolution of drought at various temporal scales is a challenging task due to the lack of long‐term measurements of surface water‐energy fluxes and states, including streamflow, radiation, evapotranspiration (*ET*), soil moisture, and snow water equivalent (SWE) (Stagge, Kohn, et al., 2015, 2017). Unlike other natural disasters (e.g., hurricanes, rainstorms, and floods), the complexity of quantifying a drought arises from the lack of a specific physical variable that can directly represent its onset, ending, and severity (AghaKouchak et al., 2015; Yang, Roderick, et al., 2018). Moreover, the definition of drought often varies with application and research interests (e.g., meteorological, hydrological, and agricultural droughts), which further contributes to difficulties associated with drought quantification (McEvoy et al., 2012; Yang, Zhang, et al., 2018; Zhang et al., 2019). A precipitation deficiency is primarily referred to as meteorological drought; a hydrological drought is commonly caused by a deficit in streamflow or low surface and groundwater levels; and an agricultural drought mainly results from low levels of soil moisture storage. Moreover, drought can occur across a variety of temporal scales (e.g., 1–48 months), which is a critical consideration for monitoring droughts, as knowledge of its multiscalar nature facilitates the quantification of the lag among meteorological, hydrological, and agricultural droughts (McKee et al., 1993; Vicente‐Serrano, Beguería, et al., 2012; Zhang et al., 2015).

Increased temperatures and atmospheric evaporative demand (Wang et al., 2012; Yin et al., 2014) is expected to reduce the amount of snowfall and SWE (Barnett et al., 2005; Margulis et al., 2016) resulting in more frequent or intense droughts (Arheimer et al., 2017; Huning & AghaKouchak, 2018; Jones & Moberg, 2003). Several studies have shown that warming processes, resulting in increased *ET* and/or soil moisture deficits, markedly affect the severity of droughts (Dai, 2011, 2013; Sheffield et al., 2012; Sheffield & Wood, 2008; van der Schrier et al., 2011; Vicente‐Serrano et al., 2010; Vicente‐Serrano et al., 2014; Zhang et al., 2017). In addition, as the atmosphere warms and less precipitation (*P*) falls as snow, the snow‐covered region (period) becomes smaller (shorter), and snowmelt occurs earlier in spring, leading to a shift in peak runoff that may increase the time lag between the peak in water availability and demand in many regions (Barnett et al., 2005). A prolonged imbalance of surface water and moisture deficiencies caused by the changing snowpack could results in more frequent hydrological and agricultural droughts, further threatening water security (Trenberth et al., 2014). Therefore, the effects of snow dynamics on water availability from global warming warrant further investigation (Jenicek et al., 2016) since snow directly affect the onset, duration, severity, and spatial extent of droughts (Staudinger et al., 2014), even without a change in *P*. Thus, considering the importance and complexity of snow in drought evolution, it is necessary to incorporate snow information into global drought monitoring.

A number of efforts have been undertaken to develop methods for drought characterization (e.g., Palmer, 1965; McKee et al., 1993; Wells et al., 2004; Sheffield & Wood, 2007; Vicente‐Serrano et al., 2010; Hao & AghaKouchak, 2013; Mu et al., 2013; Zhang et al., 2014, 2015, 2017); however, they generally neglect the effects of snow dynamics on drought evolution (Staudinger et al., 2014). Among them, drought indices are commonly used to identify drought occurrences, and thus, developing such an index for various applications is essential to drought monitoring and prediction (Zhang et al., 2015). The majority of drought monitoring studies have been conducted using either (1) the Palmer drought index, including Palmer Drought Severity Index (PDSI; Palmer, 1965) and self‐calibrated PDSI (Wells et al., 2004) or (2) a standardized index, including standardized precipitation index (McKee et al., 1993) and standardized precipitation evapotranspiration index (SPEI; Vicente‐Serrano et al., 2010), which do not incorporate the snow information needed for more robust assessments of drought in snow‐dominated regions.

The PDSI was created to measure the cumulative departure (relative to local mean conditions) of the surface moisture supply and demand (Mishra & Singh, 2010) by incorporating antecedent *P*, runoff, *ET*, and changes in soil moisture storage. However, it lacks the ability to identify drought events at multiple time scales (Wells et al., 2004; Vicente‐Serrano et al., 2010, Vicente‐Serrano, Beguería, et al., 2012; Zhang et al., 2015). Although the SPEI (Vicente‐Serrano et al., 2010) can be used to identify droughts at various time scales (Beguería et al., 2014), its main drawback relates to uncertainties in estimating potential evapotranspiration (*PET*) as a proxy for the overall water demand (Sheffield et al., 2012; Trenberth et al., 2014; Yang et al., 2006, 2007; Zhang et al., 2015).

More recently, the Standardized Moisture Anomaly Index (SZI; Zhang et al., 2015) was developed to capture the onset, ending, and severity of a multiyear drought event at a variety of time scales using water budget simulations produced with a physically based land surface model (LSM). The SZI leverages one of the strengths of the PDSI by using the moisture anomaly index (Z) as indicator of surface water deficiency or surplus. Zhang et al. (2015) demonstrated that the variability of the SZI is more consistent with observed drought evidences than that of the SPEI over water‐stressed regions because the SZI provides a more reasonable estimation of the water demand (or the climatically appropriate for existing conditions *P*, defined as $\hat{P}$) by including of *ET*, runoff, and changes in soil moisture storage (Zhang et al., 2015). By combining the water demand estimates ( $\hat{P}$) from the PDSI and the multiscalar nature of the SPEI, the SZI overcomes many weaknesses associated with the PDSI and SPEI. The shortcomings of the SZI are: (1) the need for information about many surface water‐energy components as input data (often more than many other drought indices); (2) the use of a sophisticated LSM to estimate these hydrological inputs, which can be time consuming; (3) the negligence of the effects of snow in drought characterization. In addition, it should be noted that both SPEI and SZI rely on the selection of a univariate probability distribution to standardize the indices, allowing for comparisons across climate zones (Stagge, Tallaksen, et al., 2015, 2016; Zhang et al., 2015). The choice of a probability distribution to standardize the SPEI and SZI may impart different results for the computed drought indices (Stagge, Tallaksen, et al., 2015, 2016).

The above described limitations of current drought indices highlight the need to incorporate snow information into a drought index such as the SZI to better characterize, model, and forecast droughts worldwide. Staudinger et al. (2014) proposed the Standardized Snow Melt and Rain Index, which accounts for rain and snowmelt deficits that effectively influence streamflow droughts. Although the Standardized Snow Melt and Rain Index is derived using temperature and precipitation to model snow instead of directly incorporating snow data, it provides some insights on how to upgrade the SZI for drought identification in snow‐dominated regions (Staudinger et al., 2014). To date, some analyses have been performed with this in mind by considering the effects of snowfall/snow on drought assessment (Margulis et al., 2016; Potopová et al., 2016; van der Schrier et al., 2013; Yan et al., 2014, 2016). However, the aforementioned studies were focused on the water supply instead of more broadly understanding the effect of snow on both the water supply and demand in drought quantification.

The main objective of this study is to (1) improve the SZI by incorporating snow information in both the water supply and demand using the Global Land Data Assimilation System (GLDAS; Rodell et al., 2004; i.e., establish the SZI with snow, SZI_snow_) and (2) evaluate the performance of the resulting SZI_snow_ by comparing it with SZI and observed drought evidences from multiple sources. The conceptual and technical improvement of the SZI_snow_ compared to the SZI is the key point of this work. This study contributes to the further development of drought indices by demonstrating how incorporating snow processes can improve the physical realism of drought assessment, especially in high‐latitude and high‐elevation regions covered by a deep snowpack. This work evaluates the snow states (e.g., SWE, snow depth [SNWD]) from the GLDAS product relative to observations. This study also examines different types of water‐energy responses to water deficits across a variety of regions, with particular emphasis on snow‐covered regions.

## 2 Data and Methods

### 2.1 Data Sets

#### 2.1.1 Snow Observations

The observed daily SWE and SNWD data for the western United States (includes 712 observation sites) and Alaska, United States (includes 31 observation sites) from October 1978 to September 2013 were obtained from the Snowpack Telemetry network operated by the Natural Resources Conversation Service (http://www.wcc.nrcs.usda.gov/nwcc/inventory). The observed monthly SNWD data for the Xinjiang region of China (includes 105 observation sites) from 1961–2013 were obtained from the Xinjiang Meteorological Bureau. The observed monthly SNWD and SWE data in eastern China (includes 110 observation sites) over 1980–2009 were provided by the National Meteorological Information Center of China (http://data.cma.cn/site/index.html; Wang et al., 2016). All SNWD and SWE observations were used to evaluate the performance of the snow variables from the GLDAS products.

#### 2.1.2 GLDAS Products

The GLDAS was developed by integrating ground‐based and satellite‐based observations to drive four offline LSMs (Chen et al., 2013; Rodell et al., 2004; Wang et al., 2011). Using land surface modeling and data assimilation, the widely used GLDAS provides optimal values of land surface fluxes and states for hydrometeorology studies, particularly at regional scales or over areas with limited observations. Since the GLDAS provides sufficient water‐energy variables for calculating the SZI at global scales and users do not need to run LSMs themselves, directly using GLDAS to compute the SZI could overcome the first and the second shortcomings of the SZI methods described above. Since the GLDAS product provides snowfall, SWE and snowmelt runoff at the global scale, these fields can be incorporated into the SZI.

Currently, there are two versions of the GLDAS product: GLDAS‐1 and GLDAS‐2. The GLDAS‐1 drives four LSMs at a spatial resolution of 1° × 1° from 1979 to 2017, including the Community Land Model (CLM; Dai et al., 2003), the Mosaic model (Koster & Suarez, 1994), the Noah model (Chen et al., 1996; Koren et al., 1999), and the Variable Infiltration Capacity (VIC) model (Liang et al., 1994). The GLDAS‐2 only drives the Noah model at a spatial resolution of 0.25° × 0.25° from 1948 to 2010. Both GLDAS‐1 and GLDAS‐2 data sets are available via Goddard Earth Sciences Data and Information Services Center (http://disc.sci.gsfc.nasa.gov/hydrology/data-holdings). Since the GLDAS‐2 Noah LSM product performs best in its simulation of the land water‐energy states and fluxes in most regions of the global land area (details in the supporting information Figures S1–S6), we use its monthly land surface variables to calculate the SZI and SZI_snow_ in this work.

**Figure 1:**
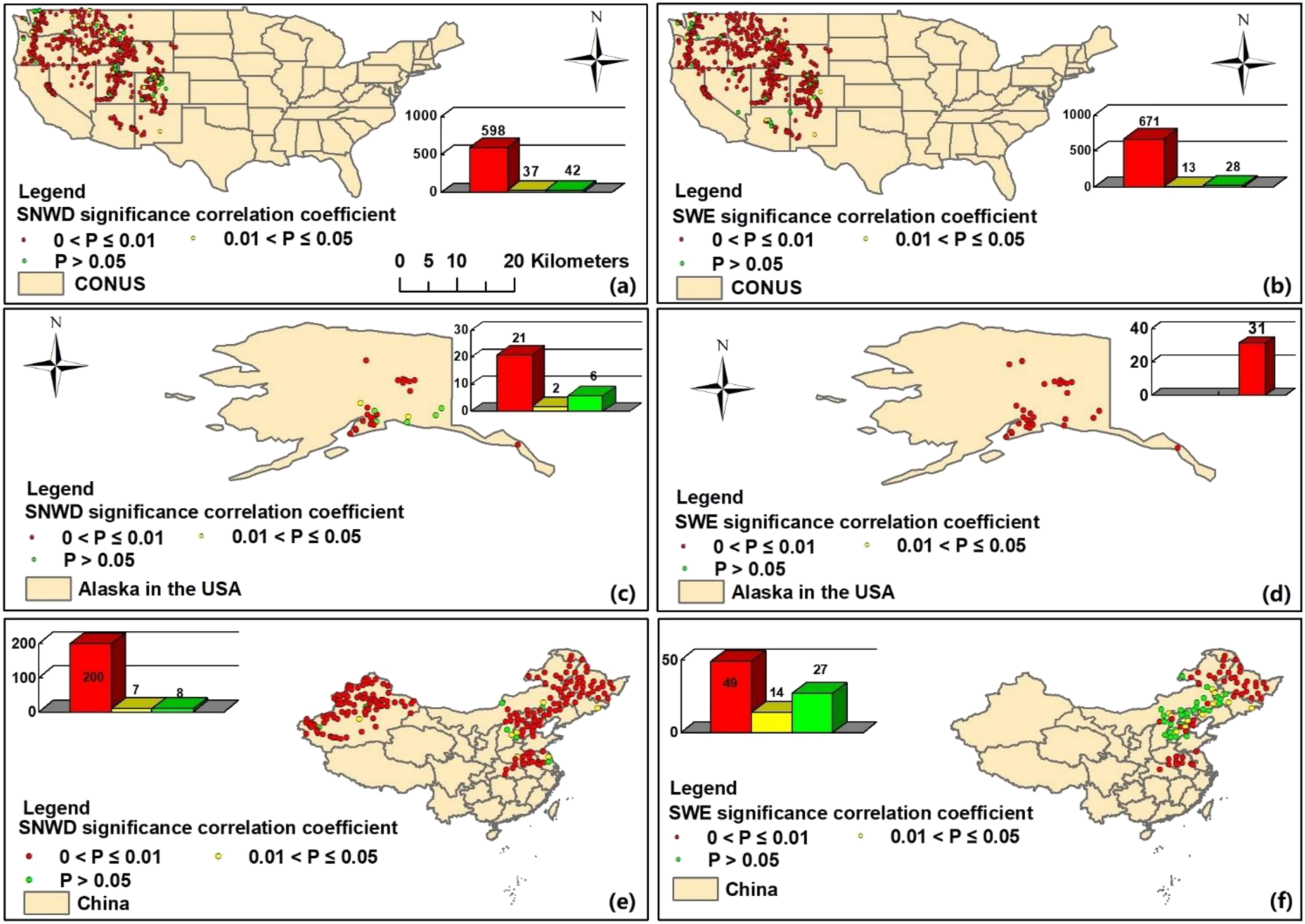
Evaluation of snowpack data simulated by the GLDAS‐2 Noah LSM using observations in the western United States (a, b), Alaska (c, d), and China (e, f). SNWD = snow depth; SWE = snow water equivalent.

In addition, the ability of GLDAS‐2 Noah LSM to accurately reproduce snow processes is an important cornerstone in the development of our drought index. To verify the reliability of its inputs for use in the SZI and SZI_snow_, SWE and SNWD from the GLDAS‐2 Noah LSM (hereafter, GLDAS‐2 refers to the GLDAS‐2 Noah LSM product) are evaluated against in situ snow observations from 958 stations over three regions (i.e., western United States, Alaska, and China). Figure 1 summarizes the statistical significance of the correlations between observed and GLDAS‐2 monthly SNWD and SWE. In the western United States, statistically significant correlations (*p* < 0.05) were found between observations of SNWD and SWE and those from GLDAS‐2 at 635 stations (94% of the total number of stations; Figure 1(a)) and 684 stations (~96% of the total stations; Figure 1(b)). The GLDAS‐2 SNWD and SWE over Alaska also show significant correlations with the observations at most stations (Figures 1(c) and 1(d)). Correlating monthly SNWD and SWE from GLDAS‐2 with observations in China (Figures 1(e) and 1(f)) indicated that statistically significant correlations exist for the SNWD case at 207 stations (96% of all 215 stations), whereas only 63 stations (or 70% of the 90 stations) exhibit significant correlations when GLDAS‐2 SWE was correlated with the observations. This likely results from biases in the meteorological forcing such as *P*, air temperature, rain‐snow partitioning, as well as model structure (e.g., single snow layer model), and parameter assumptions. In general, this demonstrates that the GLDAS‐2 reasonably represents the observed SNWD and SWE variability and serves as a viable data set for providing snow and land surface state/flux information. Nonetheless, detailed analysis explaining the underperformance of GLDAS‐2 (relative to observations) in some parts of northeastern China is provided in section 4.

The surface meteorological forcing (e.g., *P*, 2‐m air temperature), model‐simulated *ET*, and runoff/streamflow are also validated against either the observations or satellite‐based products (see Figures S1–S6). Overall, the GLDAS‐2 forcing and Noah‐simulated surface water‐energy budgets have better performance (larger correlations and smaller normalized root mean square error values) when compared with GLDAS‐1 and four LSMs simulations, suggesting a good alternative. Given the relative complexity of the LSM used to generate all hydrometeorological variables, the simulated variables could potentially introduce biases into the SZI and SZI_snow_ when comparing different sites/grids. We assume that overall effects on large basins investigated in this study are small.

#### 2.1.3 Drought Evidence Used for Evaluating the Performance

**Figure 2:**
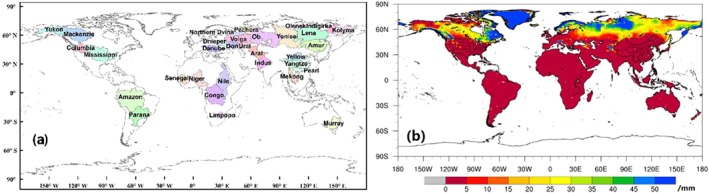
Locations of 32 large study basins a and global distribution of long‐term annual mean snow water equivalent b.

The monthly Climatic Research Unit time series Version 4.01 (CRU TS 4.01 gridded data, with a 0.5° × 0.5° resolution; http://www.cru.uea.ac.uk/cru/data/hrg/) climate data (Harris et al., 2014) and global daily *ET* product (Global Land Evaporation Amsterdam Model (GLEAM) v3.1a, at a spatial resolution of 0.25° × 0.25°; https://www.gleam.eu/) from 1980 to 2010 (Martens et al., 2017; Miralles et al., 2011) were used to determine the residual water‐energy ratio (WER; *WER* = (*P* − *ET*)/(*PET* − *ET*)) in drought conditions (for a detailed description of *WER*, see section 2.2.4; Liu et al. (2017)), wherein *PET* is calculated with the Penman‐Monteith equation (Allen et al., 1998). The observed monthly terrestrial water budget data set (including streamflow and soil water storage data) for 32 large study basins (Figure 2(a)) over the period of 1984–2006 was used to evaluate the performance of SZI and SZI_snow_ across the globe (Pan et al., 2012). Pan et al. (2012), Xu et al. (2013), and Liu et al. (2017) developed this data set using multiple observation sources (including basin‐averaged streamflow data). Basin area, location, and long‐term annual mean hydrometeorological states and fluxes of the 32 large global basins (i.e., basin area and location and long‐term annual mean hydrometeorological states and fluxes) are provided in Figure 1 and Table 1.

**Table 1:** Physical Characteristics and Long‐Term Annual Hydrologic States and Fluxes for 32 Large Basins Used to Evaluate the SZI_snow_

River basin	Drainage area (10^4^ km^2^)	Cumulative *P* (mm)	Mean SWE (mm)	Mean SNWD (mm)	Tair (°C)	SurfT (°C)
Pechora	32	525.6	47.5	268.2	−3.0	−3.5
Northern Dvina	36	598.2	37.6	196.8	1.2	0.5
Yenisei	256	443.2	31.9	229.4	−5.7	−5.4
Kolyma	64	266.0	30.2	276.8	−12.9	−12.8
Olenek	21	268.2	29.2	272.4	−13.6	−13.4
Volga	139	552.1	25.3	140.0	3.9	3.4
Ob	299	429.3	24.0	164.4	0.1	0.1
Columbia	67	594.2	23.9	94.3	6.2	6.4
Mackenzie	175	358.4	23.3	180.0	−4.3	−4.1
Lena	243	354.1	23.1	218.0	−10.0	−9.4
Indigirka	34	238.2	16.9	204.8	−16.9	−16.3
Yukon	83	244.1	14.3	103.0	−6.2	−5.7
Aral	123	246.6	9.8	52.5	9.3	10.5
Ural	24	280.6	9.0	68.5	5.5	6.2
Don	43	450.6	8.6	50.2	7.5	7.7
Dnieper	50	564.0	7.8	40.3	7.4	7.3
Danube	82	751.1	7.7	31.3	9.0	9.0
Amur	186	503.2	5.6	55.3	−1.2	−0.4
Indus	114	375.1	3.7	21.9	16.1	17.5
Mississippi	320	729.8	1.5	10.7	10.3	10.8
Yangtze	180	986.2	0.2	1.1	11.3	11.9
Yellow	80	385.6	0.2	1.1	6.9	8.2
Mekong	81	1447.1	0.1	0.4	21.3	22.0
Murray‐Darling	106	440.9	0.0	0.2	17.6	19.2
Pearl	45	1417.3	0.0	0.1	19.1	19.5
Amazon	692	2117.1	0.0	0.0	25.0	25.5
Parana	264	1170.4	0.0	0.0	21.9	22.6
Limpopo	42	520.2	0.0	0.0	21.1	22.6
Nile	308	594.9	0.0	0.0	25.5	27.6
Niger	212	648.8	0.0	0.0	27.6	29.2
Senegal	44	509.2	0.0	0.0	28.7	30.5
Congo	370	1459.5	0.0	0.0	23.9	25.0

*Note*. Tair = annual mean air temperature; SurfT = annual mean surface skin temperature; and SNWD = snow depth.

### 2.2 Methodology

#### 2.2.1 Hydrological Accounting

**Figure 3:**
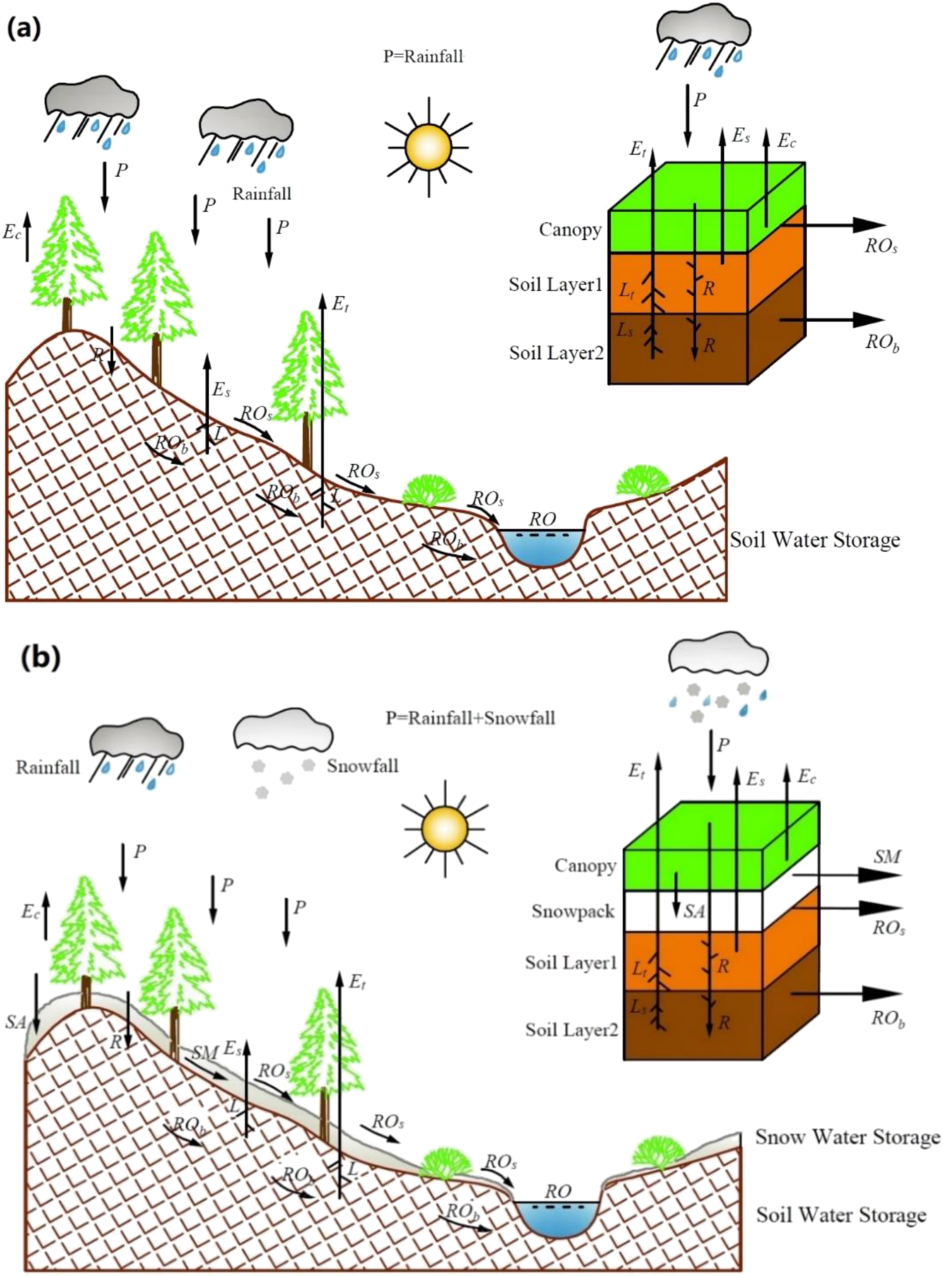
Schematic of the physical mechanisms included in the SZI (a) and SZI_snow_ (b; see section 2.2.1 for descriptions of variables). SZI = standardized moisture anomaly index.

Four monthly water budget components and their potential values were retrieved from the LSM simulations to estimate the regional water demand and carry out the hydrological accounting. These variables are runoff (*RO*), potential runoff (*PRO*), *ET*, *PET*, soil infiltration (*R*), potential soil infiltration (*PR*), soil moisture loss (*L*), and potential soil moisture loss (*PL*) as depicted in Figure 3(a). Soil moisture storage is regarded as a water reservoir in the SZI. Any changes in soil moisture storage (loss or infiltration) would influence the surface water balance (i.e., supply of water or demand of water). Contrary to the SZI, which systematically neglects snowfall as a water source and the impact of snow on surface hydrological processes (Figure 3(a)), the snow processes are introduced as another water reservoir in the water budget for SZI_snow_ (Figure 3(b)). Besides the soil moisture storage, snowpack changes (melt or accumulation) would also affect the surface water balance (i.e., supply of water or demand of water) and the drought condition quantified by SZI_snow_. As a result, the physical processes incorporated in the SZI_snow_ are more comprehensive than those in the SZI, making it applicable to a broader set of climatic regions and thereby, a broader, global community. Additionally, SZI_snow_ also accounts for the total amount of rainfall and snowfall (Figure 3(b)).

In addition to the above four water budget components and their potential values used in the hydrological accounting of SZI, two snowpack variables (snowmelt [*SM*] and SWE accumulation [*SA*]) and their potential values (potential snowmelt [*PSM*] and potential SWE accumulation [*PSA*]) are taken into account in the SZI_snow_ estimation of the total water demand (Figure 3(b)). The variables used to compute the SZI_snow_ are given by:

(1) RO=ROs+ROb+ROsmPRO=AWC−PR

where *RO*_*s*_, *RO*_*b*_, and *RO*_*sm*_ are the surface runoff, baseflow‐groundwater runoff, and snowmelt runoff, respectively; *AWC* is the available soil water holding capacity of the two layers, which is estimated as the maximum value of soil moisture over each grid cell. *ET* and *PET* are given by:

(2) $$\left\{\begin{array}{l} \mathrm{ET}=E_{b}+E_{t}+E_{i}\\ \mathrm{PET}\;\text{is}\;\text{calculated using Penman}‐\text{Monteith equation} \end{array}\right.$$

where *E*_*b*_, *E*_*t*_, and *E*_*i*_ are the bare soil evaporation, transpiration, and canopy water evaporation, respectively. The *RO*_*s*_, *RO*_*b*_, *RO*_*sm*_, *E*_*b*_, *E*_*t*_, and *E*_*c*_ are directly obtained from GLDAS‐2.

The soil profile is divided into two layers (Figure 3(b)), where the surface layer and underlying soil layer extend from 0–100 mm and 100–2,000 mm, respectively. The soil infiltration and potential infiltration are calculated as follows:

(3) $$\left\{\begin{array}{l} R=\left\{\begin{array}{l} \Delta S_{t}+\Delta S_{u}\;\Delta S_{t}+\Delta S_{u}\geq 0\\ 0\;\Delta S_{t}+\Delta S_{u}<0 \end{array}\right.\\ \mathrm{PR}=\mathrm{AWC}-\left(S_{t}+S_{u}\right) \end{array}\right.$$

where the surface layer and underlying layer available soil moisture (*S*_*t*_ and *S*_*u*_, respectively) are computed from GLDAS‐2, which in turn are used to derive their monthly changes (i.e., *ΔS*_*t*_ and *ΔS*_*u*_, respectively). The soil moisture and potential soil moisture are given by:

(4) $$\left\{\begin{array}{l} L=\left\{\begin{array}{l} 0\;\Delta S_{t}+\Delta S_{u}\geq 0\\ -\left(\Delta S_{t}+\Delta S_{u}\right)\;\Delta S_{t}+\Delta S_{u}<0 \end{array}\right.\\ \left\{\begin{array}{l} {\mathrm{PL}}_{t}=\mathrm{Min}\;\left(\mathrm{PET},\;S_{t}\right)\\ {\mathrm{PL}}_{s}=\left(\mathrm{PET}-{\mathrm{PL}}_{t}\right)\frac{S_{u}}{\mathrm{AWC}}\\ \mathrm{PL}={\mathrm{PL}}_{t}+{\mathrm{PL}}_{s} \end{array}\right. \end{array}\right.$$

where *L*_*t*_ and *L*_*s*_ are the moisture losses from the surface and underlying soil layers, respectively. *PL*_*t*_ and *PL*_*s*_ are the potential moisture losses from the surface and underlying layers. The SWE accumulation and snowmelt and their potential values are:

(5) $$\left\{\begin{array}{l} \mathrm{SA}=\left\{\begin{array}{l} 0\;\Delta \mathrm{SWE}<0\\ \Delta \mathrm{SWE}\;\Delta \mathrm{SWE}\geq 0\; \end{array}\right.\\ \mathrm{PSA}=P_{\text{snow}} \end{array}\right.$$

(6) $$\left\{\begin{array}{l} \mathrm{SM}=\left\{\begin{array}{l} ‐\Delta \mathrm{SWE}\;\Delta \mathrm{SWE}<0\;\\ 0\;\Delta \mathrm{SWE}\geq 0 \end{array}\right.\\ \mathrm{PSM}=\mathrm{SWE} \end{array}\right.$$

Lastly, *P*_*snow*_ and *∆* *SWE* are the snowfall amount and monthly change in SWE, which are derived from the GLDAS‐2 outputs. All water budget components used in this study are given in millimeter.

#### 2.2.2 Climatic Coefficients and the Climatically Appropriate for Existing Conditions *P* ( $\hat{P}$)

The $\hat{P}$ in SZI needs four water budget terms, whereas six water budget components (including SWE and snowmelt) are required for defining the water demand, ${\hat{P}}_{\text{snow}}$, for the SZI_snow_. The monthly climatic coefficients were computed as the ratios of the monthly climatic averages of the actual to potential values for *ET* (α_*j*_), soil infiltration (β_*j*_), runoff (γ_*j*_), soil moisture loss (δ_*j*_), snowpack accumulation (*ε*_*j*_), and snowmelt (φ_*j*_) as follows:

(7) αj=ETj¯/PETj¯βj=Rj¯/PRj¯γj=ROj¯/PROj¯δj=Lj¯/PLj¯εj=SAj¯/PSAj¯φj=SMj¯/PSMj¯

where *j* represents the month of the year (i.e., *j* = 1, …, 12). These ratios are taken as weighting factors or the water balance coefficients used to compute ${\hat{P}}_{\text{snow}}\;$as:

(8) $${\hat{P}}_{\text{snow}}=\alpha_{j}\mathrm{PET}+\beta_{j}\mathrm{PR}+\gamma_{j}\mathrm{PRO}+\varepsilon_{j}\mathrm{PSA}-\delta_{j}\mathrm{PL}-\varphi_{j}\mathrm{PSM}$$

#### 2.2.3 Standardizing the Moisture Anomaly Series

The difference between the actual precipitation (*P*) and ${\hat{P}}_{\text{snow}}$ is used to define the moisture anomaly *Z*_*snow*_:

(9) P=Prain+PsnowZsnow=P−P^snow

which represents the regional water deficit/surplus. Recall that in the case of SZI_snow_, *P* is equal to the sum of the total amount of rainfall (*P*_*rain*_) and snowfall (*P*_*snow*_; i.e., *P* = *P*_*rain*_+*P*_*snow*_), while the *P* in SZI only includes rainfall. Because the *SM*, *PSM*, *SA,* and *PSA* are calculated based on SWE, the SWE has a large influence on the value of *Z*_*snow*_ and thus SZI_snow_ in snowy regions.

The computed *Z*_*snow*_ values were aggregated to different time scales (i.e., 1–48 months), following the same procedure as described for the SZI by Zhang et al. (2015). We tested four three‐parameter distributions to model the *P*, *D*, *WER*, *Z*, and *Z*_*snow*_ values at different climate zones, including Pearson III, log logistic, lognormal, and general extreme values. Herein, we adopted a log‐logistic distribution for standardizing the *Z* and *Z*_*snow*_ time series to obtain the SZI and SZI_snow_ in term of what the best fit was exhibited across different climate zones. This follows the approach used in Vicente‐Serrano et al. (2010) and Zhang et al. (2015) for standardizing the values at each temporal scale. In the SZI_snow_, the average value of each standardized *Z*_*snow*_ series is zero. Negative (positive) values of SZI_snow_ indicate drier (wetter) than normal conditions (Table 2).

**Table 2:** Standardized Threshold Values for Drought and Wetness Classifications of SZI_snow_, SZI, SSI, SWSI, and SWI

Value *α* for SZI_snow_, SZI, SSI, SWSI, and SWI	Drought and wetness classification
Drought classification	
*α* < −2.0	Extreme drought
−2.0 ≤ *α* < −1.5	Severe drought
−1.5 ≤ *α* < −1.0	Moderate drought
−1.0 ≤ *α* < −0.5	Mild drought
−0.5 ≤ *α* < 0.5	Normal
Wetness classification	
0.5 ≤ *α* < 1.0	Mild wetness
1.0 ≤ *α* < 1.5	Moderate wetness
1.5 ≤ *α* < 2.0	Severe wetness
*α* ≥ 2.0	Extreme wetness

*Note*. SZI =; SSI =; SWSI =;SWI = .

#### 2.2.4 Evaluating Performance

Trenberth and Shea (2005), Adler et al. (2008), and Yin et al. (2014) suggested that water and energy are negatively correlated during drought events. This suggests that the ratio of sensible heat to net radiation (total energy supply) during a drought is larger than the normal condition, while the residual available water (*P* − *ET*) to precipitation (total water supply) is usually lower than its normal condition. Based on their theoretical analysis, Liu et al. (2017) demonstrated that the ratio of the residual available water to the residual energy (*PET* − *ET*) is relatively low (large) during drought (wet) events relative to normal conditions. Defining this ratio as *WER* = (*P* − *ET*)/(*PET* − *ET*), they proposed a method for examining the response of the surface water‐energy fluxes to drought based on *WER*. It follows that the Pearson correlation coefficient (*r*) between *WER* and SZI_snow_ that can serve as a rational evaluation criterion for the performance of SZI_snow_ as a drought indicator.

However, the *WER* proposed by Liu et al. (2017) does not consider the influence of snow accumulation or melt on the water‐energy balance. As a result, a modified version of *WER* (i.e., *WER*_*snow*_) was developed in this study by incorporating *∆SWE* as follows:

(10) $${\mathrm{WER}}_{\text{snow}}=\frac{P-\mathrm{ET}-\Delta \mathrm{SWE}}{\mathrm{PET}-\mathrm{ET}+\Delta \mathrm{SWE}}$$

To ensure that the *WER*_*snow*_ is independent of the SZI and SZI_snow_ at global scales, the variables *P* and *PET* in equation (10) for each test basin were provided by the CRU data set, while the *ET* was obtained from the remote sensing‐based GLEAM *ET* product. Because the monthly CRU data are at a uniform spatial resolution of 0.5°, the daily GLEAM *ET* data (0.25° × 0.25°) were interpolated onto monthly 0.5° × 0.5° grids to create a common resolution.

The SZI and SZI_snow_ values were also compared with observed drought evidences (streamflow as indicator for hydrological droughts and soil water storage as indicator for agricultural droughts) over 32 basins. The log‐logistic distribution is also selected to standardize streamflow (provided by Pan et al., 2012), soil water storage (provided by Pan et al. (2012)), and *WER*_*snow*_ data to compute the Standardized Streamflow Index (SSI; Vicente‐Serrano, López‐Moreno, et al., 2012), Standardized Water Storage Index (SWSI; AghaKouchak, 2014), and Standardized Wetness Index (SWI; Liu et al., 2017), respectively. Although other three‐parameter distributions, including Pearson III, lognormal, and general extreme values, were also tested for standardizing SSI, SWSI, and SWI, the test results show that the log‐logistic distribution performed the best at multiple temporal scales. To evaluate the performance of SZI and SZI_snow_ in drought identification, the Pearson linear correlation between the reference indices (SSI, SWSI, and SWI) and derived SZI and SZI_snow_ was calculated. The drought and wetness threshold levels for SZI_snow_, SZI, SSI, SWSI, and SWI are shown in Table 2 in the supporting information.

## 3 Results

### 3.1 Global Distribution of Precipitation, Snowfall, and the Water Demand

**Figure 4:**
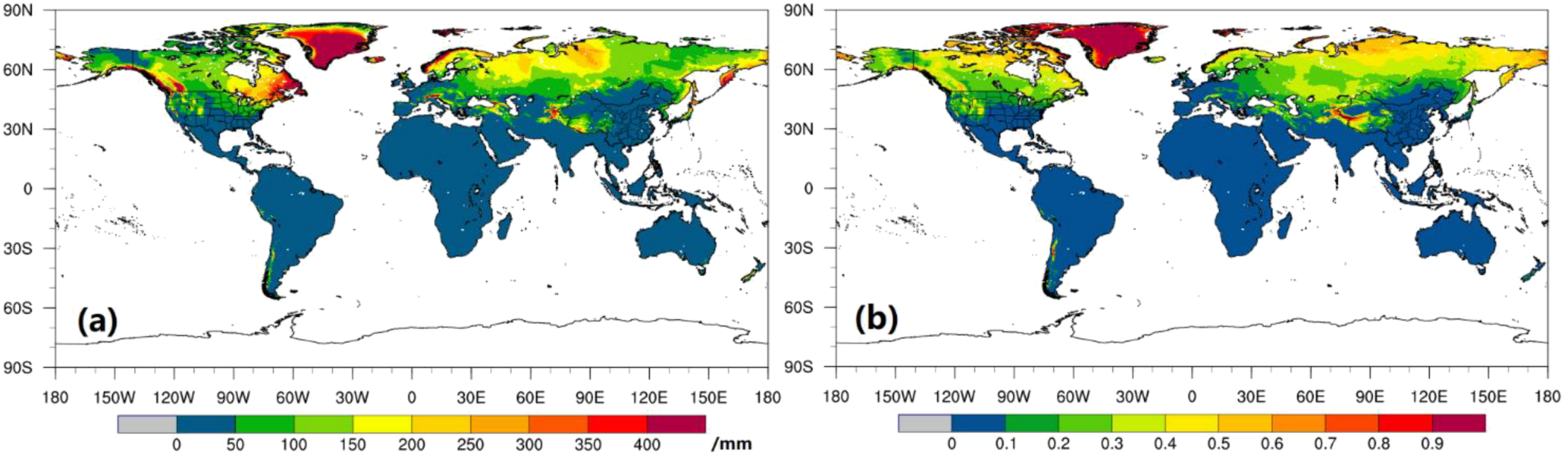
Global distribution of mean annual *P*_*snow*_ a and the ratio *P*_*snow*_/*P* b.

Since the effects of snow dynamics on both the water supply and demand were taken into account in the SZI_snow_, the differences among *P*, *P*_*snow*_, $\hat{P}$, and ${\hat{P}}_{\text{snow}}$ should firstly be examined. Snowfall (*P*_*snow*_) primarily occurs at high‐latitude and/or mountainous areas (Figure 4(a)), and the *P*_*snow*_ comprises more than 50% of *P* over polar regions and the Qinghai‐Tibetan Plateau (Figure 4(b)). Snowfall builds the snowpack on the land surface, and melt and refreezing processes modulate the snowpack during the cold season. When the land surface warms, the accumulated snowmelts and water drains into the soil increasing soil moisture and groundwater or it drains directly into the river network. This can lead to a several‐month to 1‐year lag response in the soil wetness and total water storage variability.

**Figure 5:**
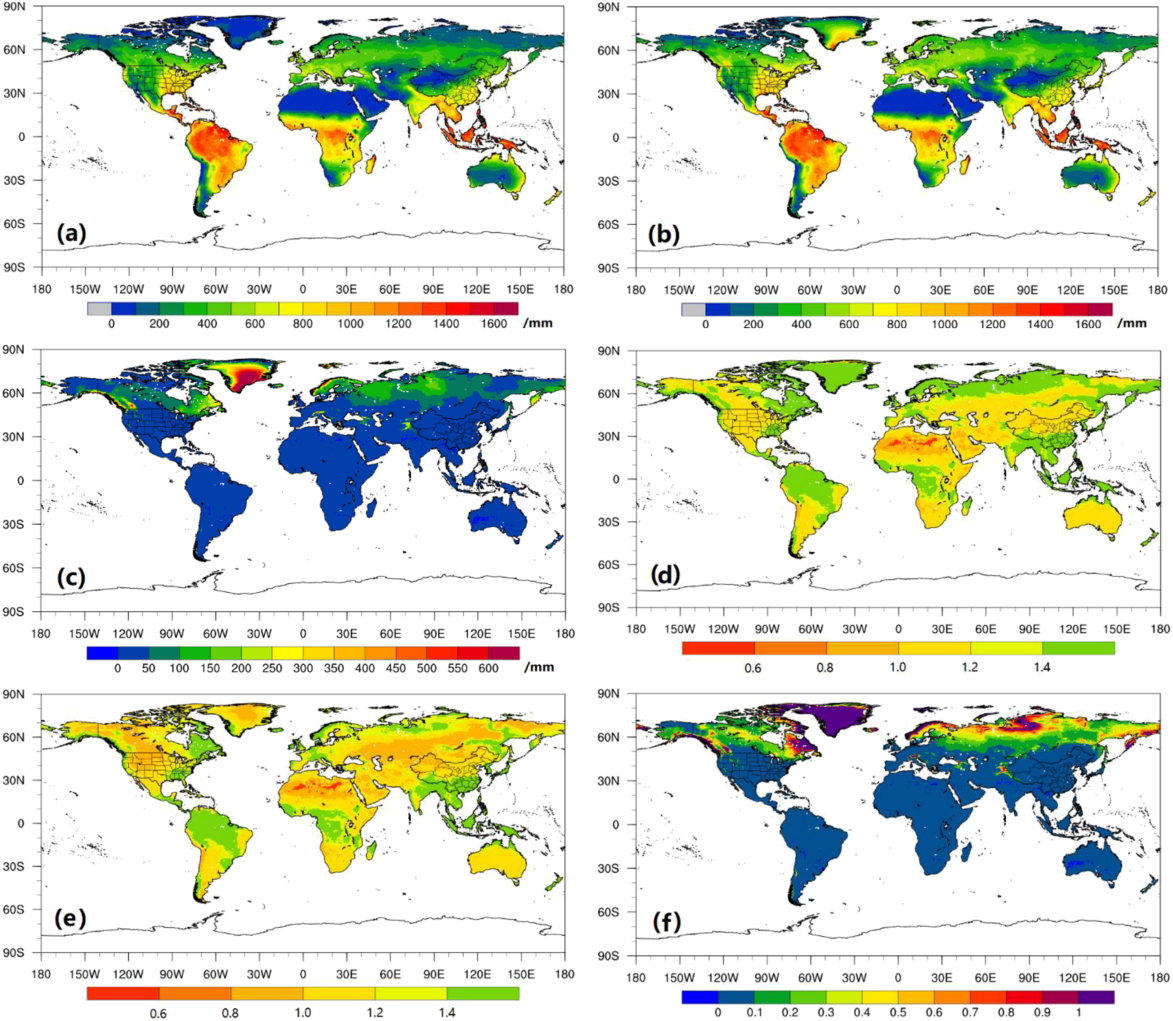
Global distribution of annual $\hat{P}$ a, annual ${\hat{P}}_{\text{snow}}$ b, and the annual difference between ${\hat{P}}_{\text{snow}}$ and $\hat{P}$ c, the ratio *P*/ $\hat{P}$ (c), the ratio *P*/ ${\hat{P}}_{\text{snow}}$ d, and the difference between *P* */* $\hat{P}$ and *P*/ ${\hat{P}}_{\text{snow}}$ e.

${\hat{P}}_{\text{snow}}$ was 100–600 mm larger than $\hat{P}$ over high‐latitude regions (Figures 5(a)–5(c)) because snowpack accumulation consumes part of the water supply (i.e., goes into storage as SWE) and enhances the water demand. The ratio of *P*/ $\hat{P}$ is ~1.0 over most regions of the globe, except in the high‐latitude areas (Figure 5(d)). In the polar region, *P* is 40% larger than $\hat{P}$, which causes a water imbalance in drought modeling and characterization as it is assumed that *P* falls as snow on the land surface and then disappears immediately. Therefore, this may degrade the performance of SZI in drought identification over different temporal scales in cold regions. By including snow accumulation and melt over cold regions, the ratio of *P*/ ${\hat{P}}_{\text{snow}}$ is closer to 1.0 than *P*/ $\hat{P}$ (Figures 5(e) and 5(f)), suggesting that SZI_snow_ is better suited for representing the water balance in snow‐covered regions than the SZI. Hence, the snow processes incorporated into SZI_snow_ result in a more generalized framework for drought assessment, which converges to SZI in regions without snow.

### 3.2 Monitoring Different Types of Droughts

To assess the performance and ability of the SZI and SZI_snow_ to monitor hydrological and agricultural droughts, the correlations between observed streamflow and soil water storage and the derived drought condition, identified by SZI and SZI_snow_, were compared at basins across the globe.

#### 3.2.1 Hydrological Drought

**Figure 6:**
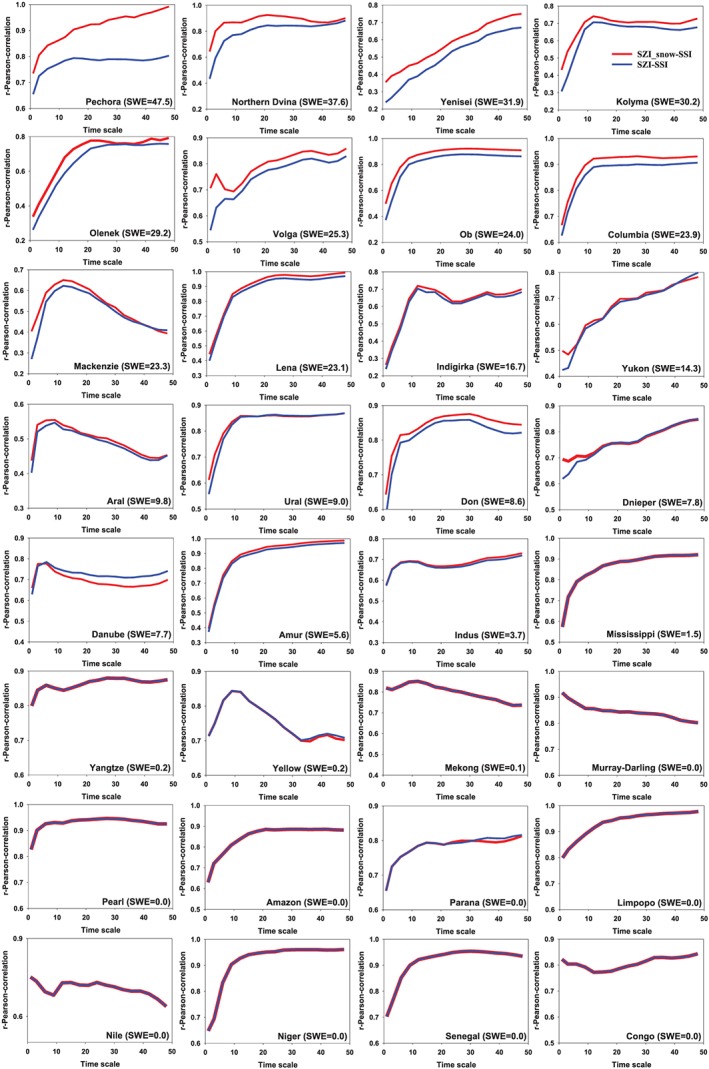
Pearson correlation coefficients for time scales from 1–48 months when SZI_snow_ and SZI are correlated with SSI at 32 global basins over 1984–2006. The SWE is long‐term annual mean value in millimeter. SWE = snow water equivalent; SSI = standardized Streamflow index; SZI = standardized moisture anomaly index.

Figure 6 shows the temporal variation of correlation coefficients between SSI and SZI/SZI_snow_ for one to 48 monthly time scales. The correlation coefficients between SZI_snow_ and SSI vary from 0.26–0.99 over the 32 basins (with an average value of 0.78). The correlation coefficients are slightly lower between SZI and SSI (0.23 ≤ *r* ≤ 0.97; average value: *r =* 0.75). However, over the basins with the highest SWE accumulation, the correlation values between SZI_snow_ and SSI are much greater than those between SZI and SSI. For basins with minimal to no snow accumulation, the correlation coefficients are almost the same. When SSI is used as a reference for a hydrological drought (Figure S7), the SZI_snow_ exhibits larger correlation coefficients with SSI than SZI does for all four snow‐dominated basins. Specifically, the performance of SZI_snow_ in identifying the 12‐month scale hydrological droughts are 12.6%, 12.5%, 19.1%, and 10.5% better than SZI at Pechora, Northern Dvina, Yenisei, and Kolyma basin, respectively. The SZI_snow_ is higher correlated because snow accumulation and melt have large impacts on the seasonal cycle and temporal variability of streamflow in such regions. Since SZI only accounts for the role of the snowpack on streamflow variation for cases when snowfall immediately produces streamflow, correlations between SZI and SSI are lower where such assumptions lead to larger deviations from the SSI. Hence, the SZI_snow_ outperforms SZI for hydrological drought identification.

#### 3.2.2 Agricultural Drought

**Figure 7:**
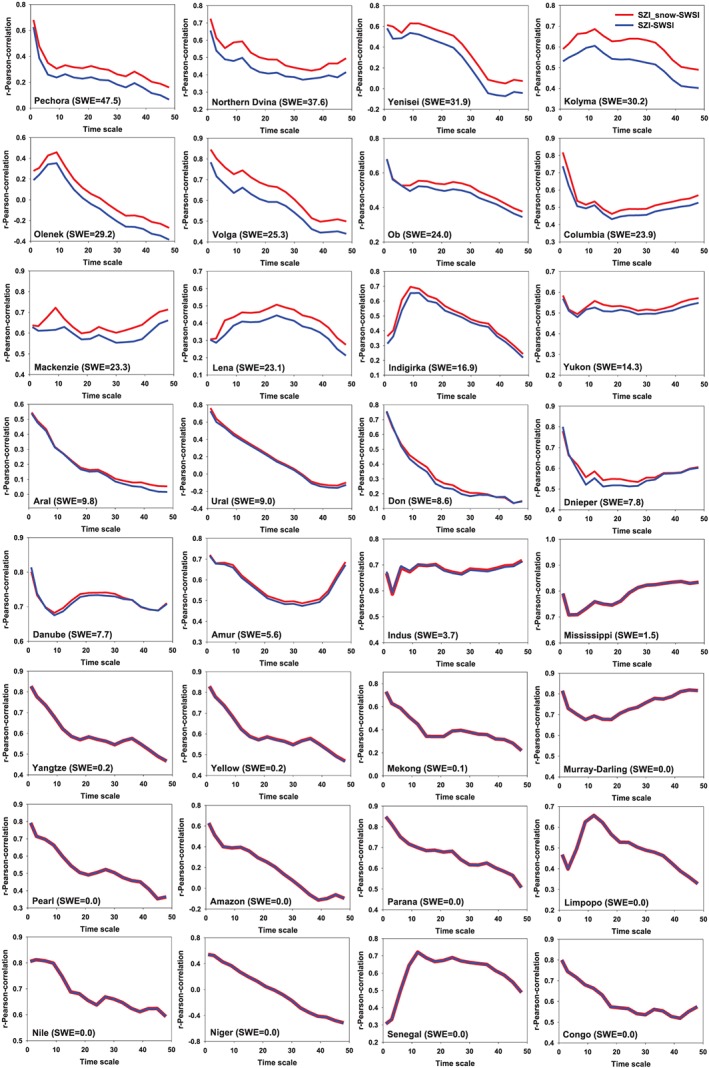
Pearson correlation coefficients for time scales from 1–48 months when SZI_snow_ and SZI are correlated with SWSI at 32 global basins over 1984–2006. The SWE is long‐term annual mean value in millimeter. SWE = snow water equivalent; SSI = standardized Streamflow index; SZI = standardized moisture anomaly index; SWSI = standardized water storage index.

The temporal variation of correlation coefficients between SWSI and SZI, as well as those between SWSI and SZI_snow_ for the same 32 basins is illustrated in Figure 7. The average correlation over all of the basins is comparable for the SZI and SZI_snow_ cases (i.e., 0.46 vs. 0.50, respectively). However, for those basins receiving large amounts of SWE, the correlation coefficient associated with SZI_snow_ is higher than that of the SZI. When the SWSI is used as a reference (Figure S8), SZI_snow_ outperforms SZI for quantifying agricultural droughts. Specifically, the correlations between SZI_snow_ and SWSI are 31.4%, 18.6%, 14.3%, and 13.2% higher than those between SZI and SWSI at the 9‐month scale over Pechora, Northern Dvina, Yenisei, and Kolyma basin, respectively.

Moreover, it should be noted that both the SZI and the SZI_snow_ not only can be calculated based on regional or basin averages but also for individual grid cells. To demonstrate this, we show regions experiencing drought conditions as defined in Table 2 over the heavily snow‐influenced Lena, Mackenzie, Ob, Volga, and Yenisei basins (Figure S9). The results confirm our conclusions based on correlation analysis—the SZI_snow_ better characterizes hydrological and agricultural droughts in high‐latitude and/or high‐elevation regions with a deep snowpack. Also, the SZI_snow_ identifies the water‐energy residual during drought events (which is one of the most important indicators of multiple categories drought events) in all five basins at grid cell level.

### 3.3 Global Evaluation Using the SWI

**Figure 8:**
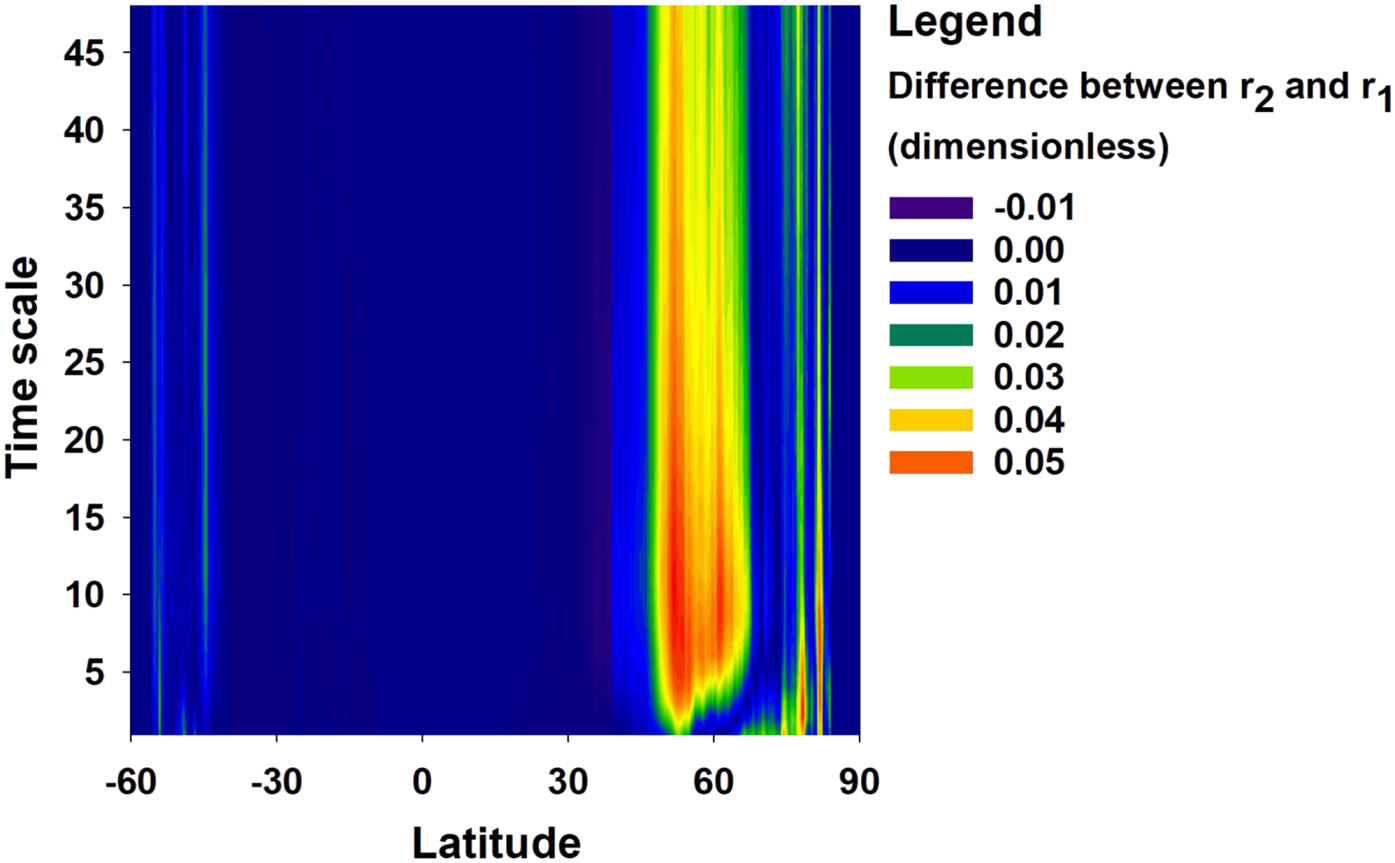
Difference between two correlation coefficients (*r*_2_ − *r*_1_), where *r*_2_ is the correlation between SZI_snow_ and SWI and *r*_1_ is the correlation between SZI and SWI. The array is shown for latitudes 60^o^S–90^o^N and 1‐ to 48‐month time scales.

To further understand the effects of including snow dynamics in drought modeling at the global scale, the latitudinal and temporal variations of the differences between the correlation coefficients between SZI_snow_ and SWI are compared to the correlation coefficients between SZI and SWI, which is taken as reference in this case (Figure 8). The difference between the two sets of correlation coefficients are generally positive, and the highest values were found between 50^o^N–65^o^N, suggesting that SZI_snow_ performs better than SZI, particularly in this 15^o^ latitudinal range. In contrast, the correlation coefficient values are nearly identical in the mid‐ and low‐latitude regions where snow impacts the drought characterization less. Although performance depends on latitude, SZI_snow_ shows the largest improvements over SZI at 3‐ to12‐month scales, which is largely attributed to the fact that *WER* is most sensitive to snowmelt/accumulation within one water year (but insensitive to snow dynamics shorter than the 3‐month scale).

**Figure 9:**
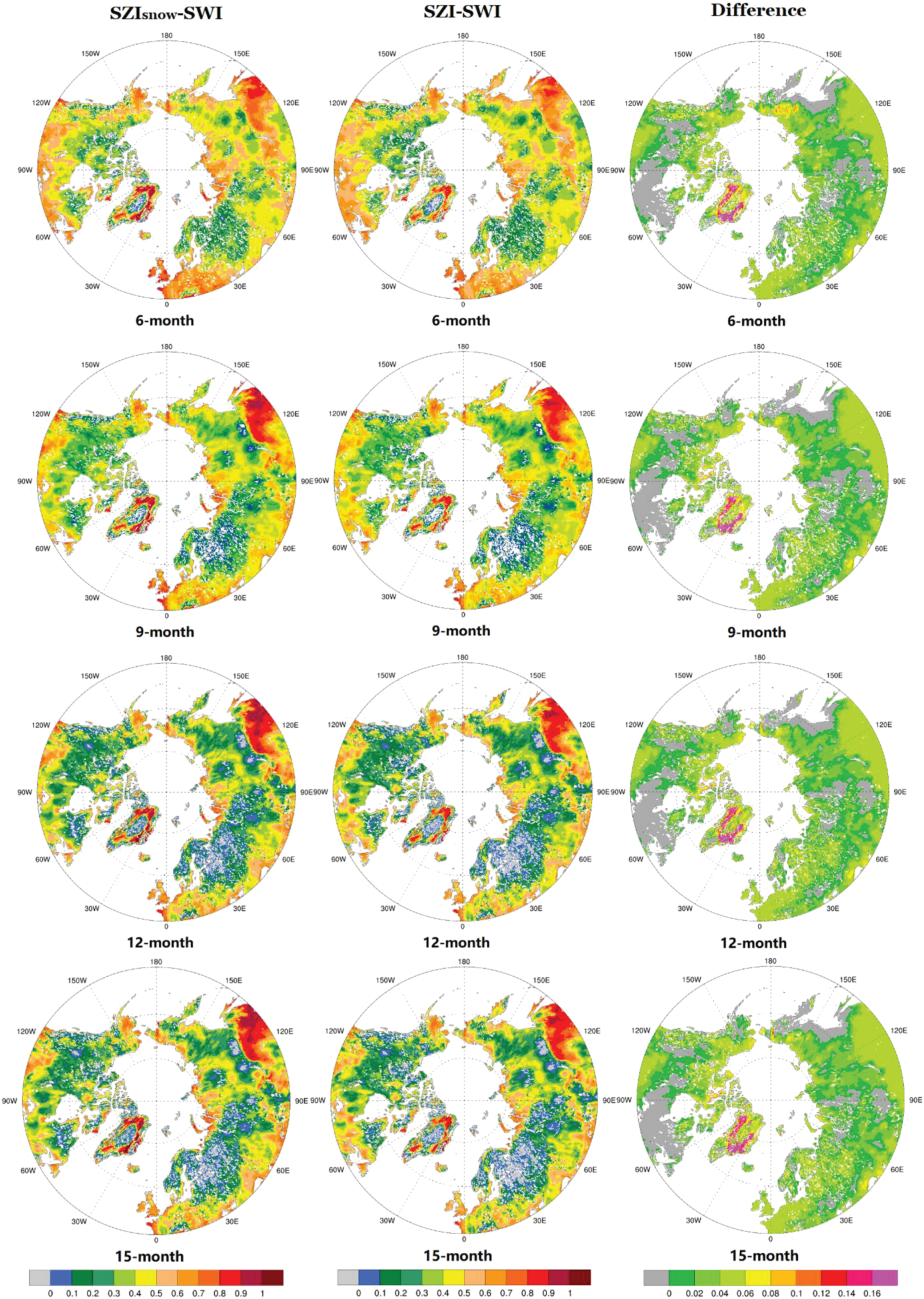
Spatial distribution of correlation coefficients between SZI_snow_ and SWI (left column) and between SZI and SWI (middle column), and the differences between the two maps (left column ‐ middle column) in the Arctic for various temporal scales. SZI = standardized moisture anomaly index; SWI = standardized wetness index.

Maps of correlations between SZI_snow_ and SWI (left column), correlations between SZI and SWI (middle column), and the differences between them (right column) in the Arctic region are shown in Figure 9 over a variety of temporal scales. The correlations vary spatially for the 6‐, 9‐, 12‐, and 15‐month time scales (from top to bottom in Figure 9). Generally, the differences between correlations are positive for most Arctic regions across all time scales, highlighting the value of considering snow dynamics in SZI_snow_. As the time scale increases from 6–12 months, this improvement also increases (Figure 9). Overall, the addition of snow processes improves upon SZI performance across the Arctic, particularly at the 6‐ to 12‐month time scales, although this improvement varies spatially across the time scales considered here (Figure 9).

## 4 Discussion

### 4.1 Advantages of SZI_snow_

The development of drought indices for varied applications is essential to drought prediction/mitigation and water resources management (Liu et al., 2015, 2016). However, it is still difficult to establish a universal drought index that can monitor and identify all types of droughts. Nonetheless, the SZI_snow_, resulting from the incorporation of snow dynamics into the SZI, addresses deficiencies in the SZI as well as the PDSI and SPEI that are important for identifying, monitoring, and quantifying agricultural and hydrological droughts specifically in those climatic regions with distinct snowmelt and snow accumulation/wet seasons. Because the SZI_snow_ requires information about *P*, SWE, *RO*, *ET*, and changes in soil water storage, which can be easily obtained from LSMs through Land Data Assimilation Systems or Coupled Model Intercomparison Project, it is suitable for regional comparisons using distributed or gridded hydrometeorological data sets without any additional information required. It should be noted that the performance of the SZI_snow_ is equal to the performance of the SZI in areas that are snow free.

### 4.2 Limitations of SZI_snow_

The main limitation of SZI_snow_ is that its computation is more complex and difficult than the computation of the standardized precipitation index, the SPEI, and the SZI as it adds several variables associated with snowmelt and accumulation processes. Although the operational application of the SZI_snow_ is important for improvement of mitigation and disaster reduction strategies related to droughts, at its current stage, the SZI_snow_ cannot yet be used for operational application due to its complexity. Therefore, a collaboration between our researchers and operational agencies/centers (e.g., United States Drought Monitor, China Meteorological Administration) to test operational feasibility of the SZI_snow_ is needed in the future. Another limitation of the SZI_snow_ is that its calculation requires long‐term climatic and hydrologic records, which makes it unsuitable for short‐term drought identification.

Moreover, because the establishment of SZI_snow_ is based on GLDAS or other similar LSMs output, the performance and quality of these models directly determines the accuracy of SZI_snow_ in drought characterization. Since the GLDAS or LSM inevitably have uncertainties including forcing data errors, model structure deficiencies, and model parameters errors, an urgent issue that needs to be addressed in the future becomes how to minimize the effects of the uncertainties associated with input data for SZI_snow_. Despite such limitations, the SZI_snow_ provides a better tool for monitoring water resources (dry and wet spells), particularly in high‐latitude and high‐altitude regions with thick snowpack. Although the SZI_snow_ is a physically based multiscalar and multicategory drought index, averaging across a variety of topographic features over large areas, the uncertainty of precipitation input, and so forth can potentially cause unrealistic water budgets on large scales and introduce systematic biases in drought monitoring.

### 4.3 Uncertainties of Snow Data From GLDAS Products

The representation of the snow processes in the GLDAS product is crucial because snow has a large influence on water‐energy fluxes on the land surface, thereby affecting the accuracy of drought identification. For instance, the high albedo of snow determines the amount of solar radiation absorbed by the land surface, which influences the turbulent exchanges of water and energy between the land and the atmosphere. The hydrological application of GLDAS also depends largely on the accurate representation of snow processes because the hydrology of cold regions is heavily influenced by snow accumulation and melt (Bales et al., 2006). However, uncertainty remains with respect to the representation of snow on the ground in the GLDAS product, as evidenced by the low correlations between the SWE (or SNWD) GLDAS fields and observations in certain regions (Broxton et al., 2016).

**Figure 10:**
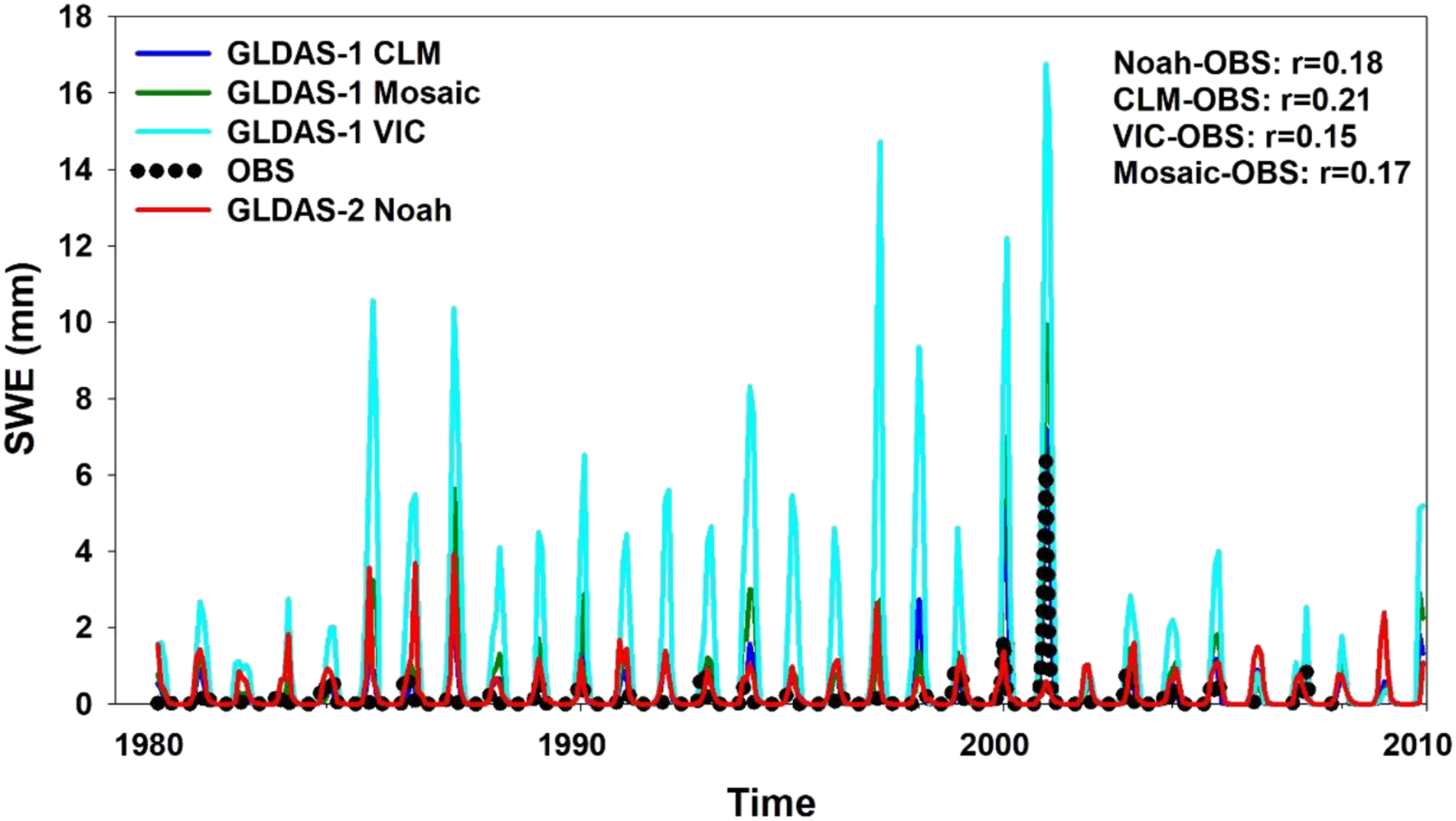
Comparison of monthly‐averaged SWE OBS and fields from GLDAS‐2 Noah LSM and GLDAS‐1 CLM, mosaic, and VIC LSMs over northeastern China for the green dots in Figure 1(f) (the simulations and observations at different points are averaged to a mean value). CLM = community land model; GLDAS; SWE = snow water equivalent; OBS = observations; VIC = variable infiltration capacity; LSM = land surface model.

Although we showed that the GLDAS‐2 provides reasonable estimates of SWE and SNWD across several regions of the world (Figure 1), large uncertainties exists in some parts of northeastern China. A comparison between the SWE observations and GLDAS fields from four LSMs over northeastern China (the green dots in Figure 1(f)) is shown in Figure 10. For these locations, the four LSMs do not capture the temporal variations of the SWE observations well since correlation coefficients are small (0.15 ≤ *r* ≤ 0.21). The long‐term annual mean observed SWE is 1.26 mm, while the SWE from GLDAS‐2 Noah and GLDAS‐1 CLM, VIC, and Mosaic are 3.39, 3.14, 13.66, and 5.33 mm, respectively, indicating large uncertainties relative to the observations and among the various LSM estimates. The SWE estimates from the four LSMs are substantially higher than observed. The reason for these uncertainties remains unclear and needs further investigation from many aspects such as forcing data, model structures, and model parameters. Besides, there are uncertainties and biases associated with the observed SWE data as well (Meyer et al., 2012). Nonetheless, a large spatial disparity exists between the observations and GLDAS SWE fields from the four LSMs.

**Figure 11:**
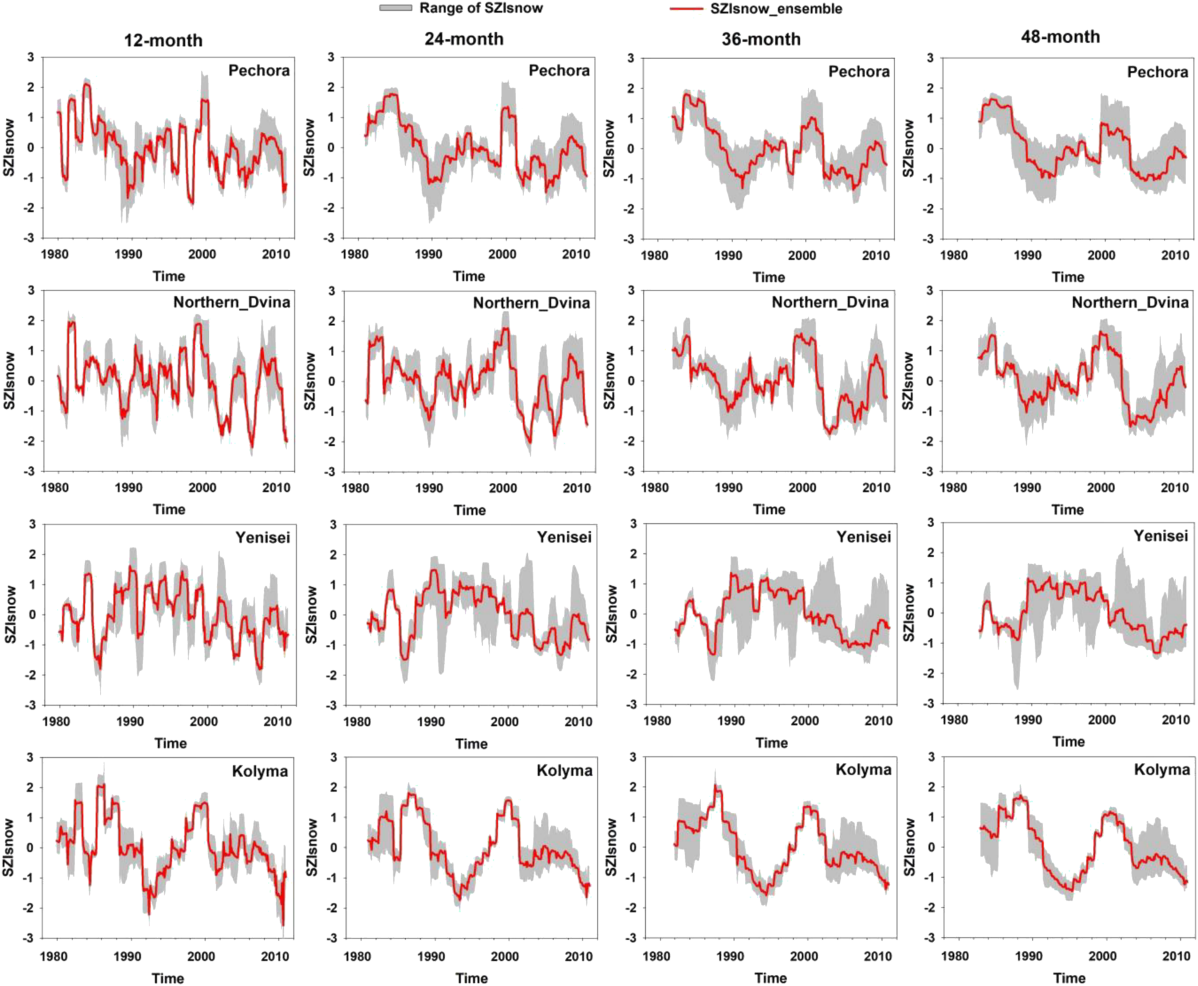
Basin‐averaged SZI_snow_ computed using the mean (red lines) and range of SZI_snow_ values (gray shading) from the individual LSMs at 12‐, 24‐, 36‐, and 48‐month scales. SZI = standardized moisture anomaly index; LSM = land surface model.

In practical application, not only SWE variability but also its amount has large impact on the SZI. Generally, snow amount measurement has errors due to blowing snow caused by wind, vegetation density, and topographic slope effect. For simulated and satellite retrieved snow amount, there are large uncertainties and errors as snow dynamics included in LSMs is not well understood and remote sensing techniques is limited in snow cover conditions. Besides SWE, an additional measure can be used is SNWD (volume). In general, SWE can be derived from SNWD when snow density is known. However, snow density varies with snow age, which is a very difficult to measure. Such a conversion brings an extra challenge when SNWD is used. Nevertheless, it should be noted that both snow variability and amount uncertainties (e.g., SWE, SNWD) have a large impact on SZI calculation and analysis and need to be further investigated in the future. We simply used the GLDAS‐1 products from three models (CLM2.0, Mosaic, and VIC4.0.4) and GLDAS‐2 (Noah) to quantify the uncertainty associated with SZI_snow_ since snowfall and SWE are used in the calculation. Figure 11 provides a comparison of the mean and range of SZI_snow_ values calculated across the four GLDAS products for the selected snow‐dominated basins (rows) at 12‐, 24‐, 36‐, and 48‐month time scales (columns). Using the GLDAS products, large uncertainties exist across all four basins (Figure 11). As the time scales increase, the uncertainties become larger, again, depending on the basin and time scale (Figure 11).

Despite that the GLDAS products have relatively large uncertainties when the result from low quality meteorological forcing data (in particular precipitation data) are used to run LSMs, hydrological, and agricultural studies over regions that lack observations still would benefit from GLDAS products. We recognize that the uncertainty associated with *P*_*snow*_ and *P*_*rain*_ data influences the results of SZI_snow_. However, despite uncertainties in the GLDAS snow simulation, theoretically, the accuracy of the input water budget components does not influence the conceptual or technical improvement of the SZI_snow_, since the impact of snow dynamics is considered for both water supply and demand in our drought characterization. As soon as additional GLDAS and reanalysis products including satellite retrievals become available, a multisystem and multimodel SZI_snow_ along with uncertainty estimates will be calculated to enhance its robustness and reliability. This ongoing work will be reported in the future.

## 5 Conclusions

The SZI_snow_, as introduced in this work, overcomes the limitations of the SZI by using components of the water‐energy budget from the GLDAS‐2 Noah LSM to incorporate the influence of snow on both the water supply and demand in drought characterization. Although we computed the SZI_snow_ using GLDAS products, the general framework for calculating the drought index from other data sets is possible given that reasonable estimates of the required water and energy budget variables are available. Furthermore, the SZI_snow_ can be applied at multiple spatial (i.e., grid cell to global scales) and temporal scales (1 to 48 months) as demonstrated herein. Therefore, this index provides the community not only with a global drought identification data set, but also a complementary methodology for characterizing and monitoring hydrological and agricultural droughts across both snow‐covered and snow‐free regions.

The application of SZI_snow_ to snow‐covered regions is particularly important given that the snow impacts the surface water balance and thus affect the onset, duration, intensity, and spatial extent of drought at varied time scales. In this study, we demonstrated the usefulness of SZI_snow_ for drought monitoring at multiple temporal scales across the globe, particularly in snow‐covered basins. Except for some parts of northeastern China, the GLDAS‐2 Noah LSM snowfields performed well across most regions considered relative to in situ SWE and SNWD observations. This indicates that the GLDAS‐2 Noah LSM product is qualified for use in global application of the SZI_snow_. In the 32 basins specifically examined herein, the SZI_snow_ had larger correlations with the observed changes in hydrological and agricultural droughts than the SZI for the basins with a deep snowpack.

Although the SZI_snow_ requires more information than the SZI to additionally account for snowpack accumulation and melt processes in the water budget, this additional complexity yields meaningful improvements in snow‐covered regions. As demonstrated herein, the more that a basin is influenced by snow, the more worthwhile it becomes to complement the SZI with the SZI_snow_. In addition, it is not surprising that the SWI is more consistent with the SZI_snow_ than the SZI over high‐latitude and high‐elevation cold regions, suggesting the imporoved value of the SZI_snow_ for identifying, monitoring, and characterizing drought events. The theoretical improvement that the SZI_snow_ has over SZI occurs since the former accounts for snow processes in drought identification, and this should be reflected assuming that reasonable values are input for the water‐energy budget (as demonstrated with GLDAS‐2 Noah LSM fields herein).

Finally, we note that most of the existing Drought Early Warning Systems (DEWSs) focus on the hazard component of drought monitoring, without accounting for snow processes (e.g., Wilhite et al., 2000; Hao et al., 2014, WMO and GWP, 2016, and therein). The existing DEWSs can benefit from incorporating snow information especially in snow‐dominated regions. This paper offers a possible pathway forward to improve exiting DEWSs through incorporating SZI_snow_.

## Supporting information



Supporting Information S1
