# Supplementary material for: A Framework for Global Multicategory and Multiscalar Drought Characterization Accounting for Snow Processes
Source: Water Resour Res. 2019 Nov 19;55(11):9258–78. doi: 10.1029/2019WR025529 (PMC6988450; doi:10.1029/2019WR025529)
Supplement: Supplementary file 1 — Supporting Information S1 [file WRCR-55-9258-s001.doc]

Water Resources Research

Supplementary Information for

**A framework for global multi-category and multi-scalar drought characterization accounting for snow processes**

Baoqing Zhang^1,2^ • Youlong Xia^3^ • Laurie S. Huning^4,5^ • Jiahua Wei^2*^ • Guangqian Wang^2*^ • Amir AghaKouchak^4,5^

1. Key Laboratory of Western China's Environmental Systems (Ministry of Education), College of Earth and Environmental Sciences, Lanzhou University, Lanzhou, Gansu, China

2. State Key Laboratory of Hydroscience and Engineering, Department of Hydraulic Engineering, Tsinghua University, Beijing, China

3. I. M. Systems Group at Environmental Modeling Center (EMC), National Centers for Environmental Prediction (NCEP), College Park, Maryland, USA

4. Department of Civil and Environmental Engineering, University of California, Irvine, California, USA

5. Center for Hydrometeorology and Remote Sensing, University of California, Irvine, California, USA

**Contents of this file**

Figures S1 to S9.

**Introduction**

This supplementary information includes descriptions of the Global Land Data Assimilation Systems (GLDAS) products and a verification of their reliability relative to multiple types of observation, and additional data and analysis.

**Description of GLDAS products and Data Verification**

Currently, there are two versions of GLDAS product: GLDAS-1 and GLDAS-2. The GLDAS-1 uses four LSMs at a spatial resolution of 1° × 1° from 1979 to 2017, including the Community Land Model (CLM) (Dai et al., 2003), the Mosaic (MOS) model (Koster & Suarez, 1994), the Noah model (Chen et al., 1996; Koren et al., 1999), and the Variable Infiltration Capacity (VIC) model (Liang et al., 1994). The GLDAS-2 only utilizes the Noah model at a spatial resolution of 0.25° × 0.25° from 1948 to 2010. Although these LSMs can provide terrestrial water and energy fluxes and states, the accuracy of surface water and energy variables must be evaluated before being practically applied. In this study, we evaluate both forcing data and model output of four LSMs in GLDAS-1 and the GLDAS-2 Noah LSM relative to climatic observations from CRU TS 4.01, global evapotranspiration (*ET*) data from GLEAM, and streamflow data from Pan et al. (2012) at 32 global large basins.

Because precipitation (*P*) and air temperature (*T*) are two important forcing fields in GLDAS LSMs, these two variables were firstly evaluated with the CRU TS 4.01 datasets. As shown in Figure S1, both *P* and *T* in GLDAS-1 and GLDAS-2 are consistent with CRU TS 4.01 datasets in most regions of the world, except for Greenland and some African areas, indicating that the forcing data in the GLDAS products perform relatively well at the global scale. It should be noted that the Pearson correlation coefficient (*r*) for *P* between GLDAS-2 Noah LSM and CRU TS 4.01 (mean *r* value is 0.85) are apparently higher (7.6%) than those for GLDAS-1 (mean *r* value is 0.72) as GLDAS-2 uses the Princeton surface meteorological forcing (Sheffield et al., 2006). Instead GLDAS-1 uses a combination of NCEP’s Global Data Assimilation Systems (GDAS), disaggregated Climate Prediction Center Merged Analysis of Precipitation (CMAP), and Air Force Weather Agency (AFWA) radiation datasets. In addition, the *T* from the GLDAS-2 Noah LSM also agrees better with the CRU TS 4.01 dataset (mean *r* value is 0.99) than the GLDAS-1 product (mean *r* value is 0.91) does. The global distribution of Normalized Root Mean Square Error (NRMSE) for *P* and *T* between GLDAS and CRU TS 4.01 are shown in Figure S2. Figure S2 confirms the conclusions from Figure S1 that that the forcing data of GLDAS falls within a reasonable range for most regions globally, in particular for GLDAS-2 *P* and *T* (larger correlation and smaller NRMSE).

To further evaluate the model output of the GLDAS products, the *ET* and streamflow, which are two crucial variables in determining the water demand metric in the SZI and the SZI_snow_, were validated with the GLEAM *ET* product (based on satellite observations) and the streamflow data in 32 global basins from Pan et al. (2012), respectively. Figure S3 shows the global distribution of correlations between GLDAS *ET* and GLEAM *ET*. The simulated *ET* in both GLDAS-1 and GLDAS-2 agree well with the satellite observed *ET* (GLEAM) over most regions of the world except for some regions with large forcing errors (e.g., Greenland, Africa) and model deficiencies (e.g., South American forest regions). The average *r* value is 0.75 for *ET* between GLDAS-2 Noah LSM and GLEAM, which is slightly higher than that for the GLDAS-1 CLM (mean *r* is 0.73), MOS (mean *r* is 0.73), Noah (mean *r* is 0.74), and VIC (mean *r* is 0.73) LSMs. The global distribution of the NRMSE for *ET* between GLDAS and GLEAM is included in Figure S4. Overall, simulated ET products are quite a good and NRMSE lies within a reasonable range. The performance of transpiration (*E_t_*), bare soil evaporation (*E_b_*), and canopy water evaporation (*E_i_*) from the GLDAS-2 Noah LSM are exhibited in Figure S5. Figure S5 shows that the averaged *r* for *E_t_*, *E_b_*, and *E_i_* between GLDAS-2 Noah LSM and GLEAM are 0.70, 0.61, and 0.67, respectively, which indicates that the simulated *ET* fluxes in GLDAS-2 Noah LSM are generally of high quality over most parts of the global land area. Figure S6 exhibits the boxplots of the correlations and NRMSE between the GLDAS streamflow and the streamflow data from Pan et al. (2012) over the 32 basins. Figure S6 illustrates that the *r* values for GLDAS-2 Noah LSM range from 0.48 to 0.96 (average = 0.77), which are higher than those for the GLDAS-1 CLM (range from 0.37 to 0.91, average = 0.69), MOS (range from 0.14 to 0.78, average = 0.50), Noah (range from 0.29 to 0.88, average = 0.63), and VIC (range from -0.01 to 0.85, average = 0.51) LSMs. The averaged NRMSE values for GLDAS-2 Noah LSM are generally lower than those for the four LSMs in GLDAS-1.

Overall, Figures S1-S6 demonstrate that the GLDAS-2 Noah LSM performs the best out of the GLDAS products in simulating the land water-energy states and fluxes in most regions of the globe (larger correlation and smaller NRMSE with observations). Better performance of GLDAS-2 results from its use of more accurate and consistent surface meteorological forcing data, as well as the model upgrade (GLDAS-1 uses version 2.7.1 of Noah model and GLDAS-2 used version 3.3 of Noah model). Therefore, we calculated the SZI and SZI_snow_ using the GLDAS-2 Noah LSM at a spatial resolution of 0.25° × 0.25° from 1948 to 2010 in this work.

**References**

The citations in this Supporting Information (SI) can be found in the reference list in the main article.


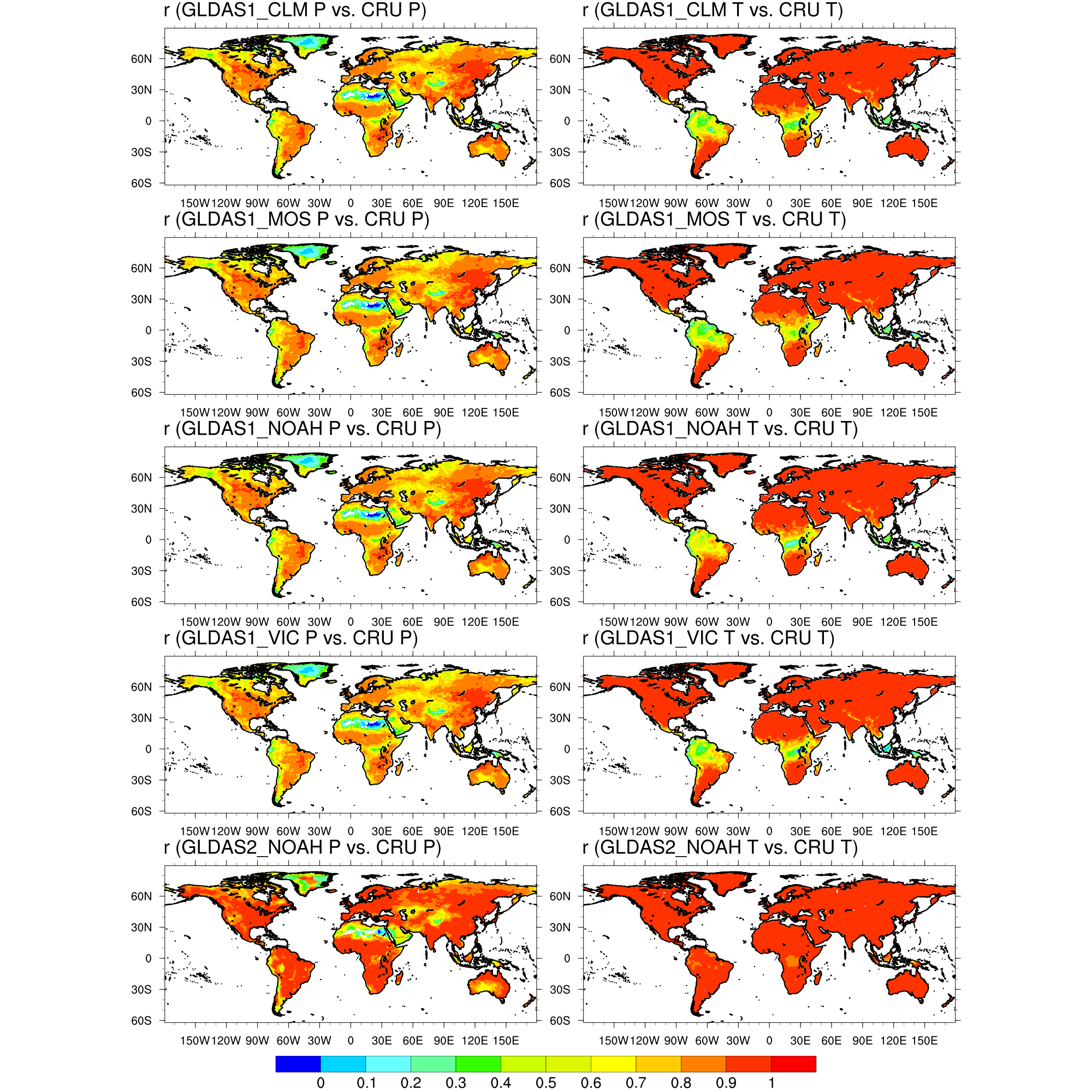


**Figure S1.** Global distribution of correlations between GLDAS and CRU TS 4.01 for *P* (left column) and *T* (right column). The correlation is calculated for a 1979–2010 period.


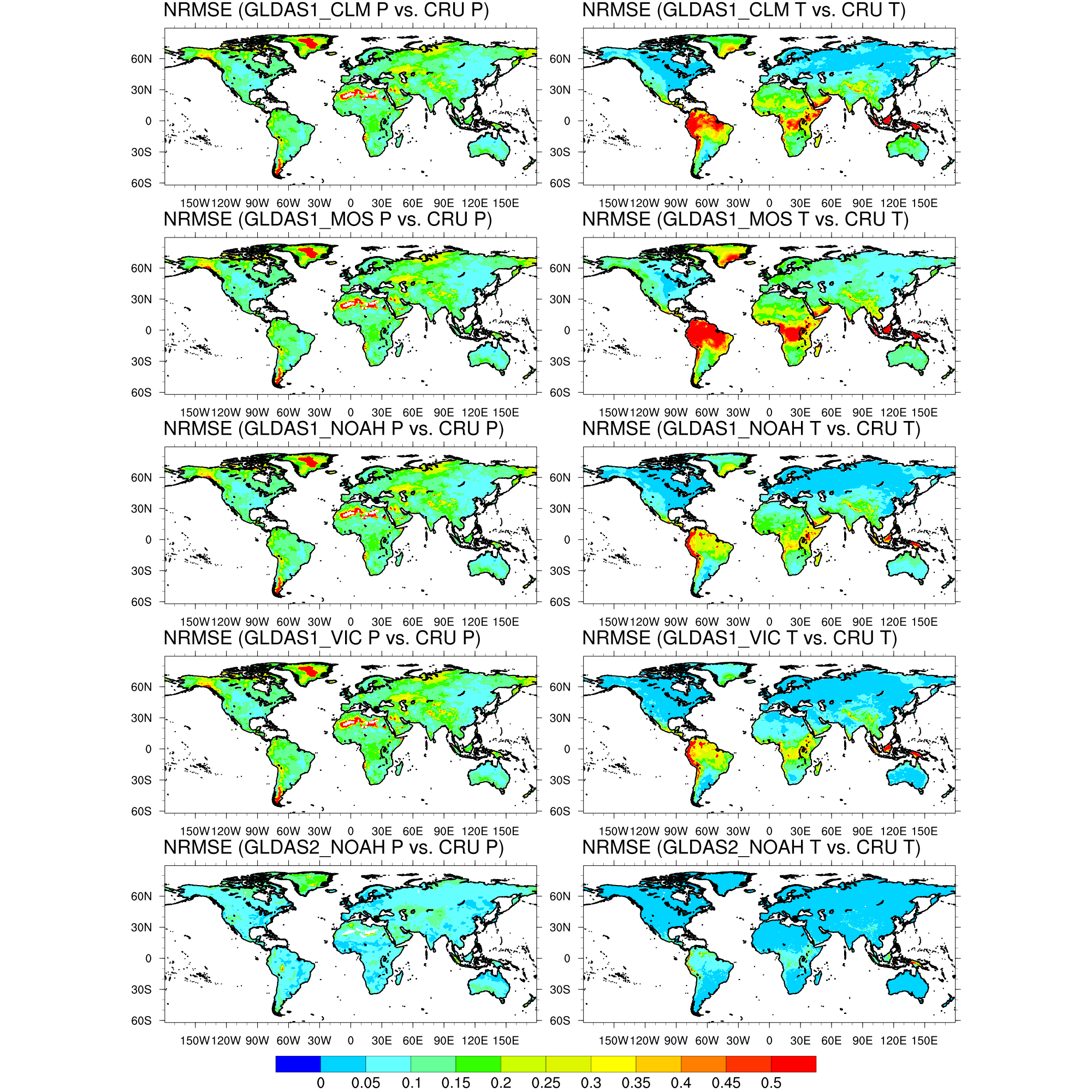


**Figure S2.** Global distribution of NRMSE between GLDAS and CRU TS 4.01 for *P* (left column) and *T* (right column) over 1948–2010.


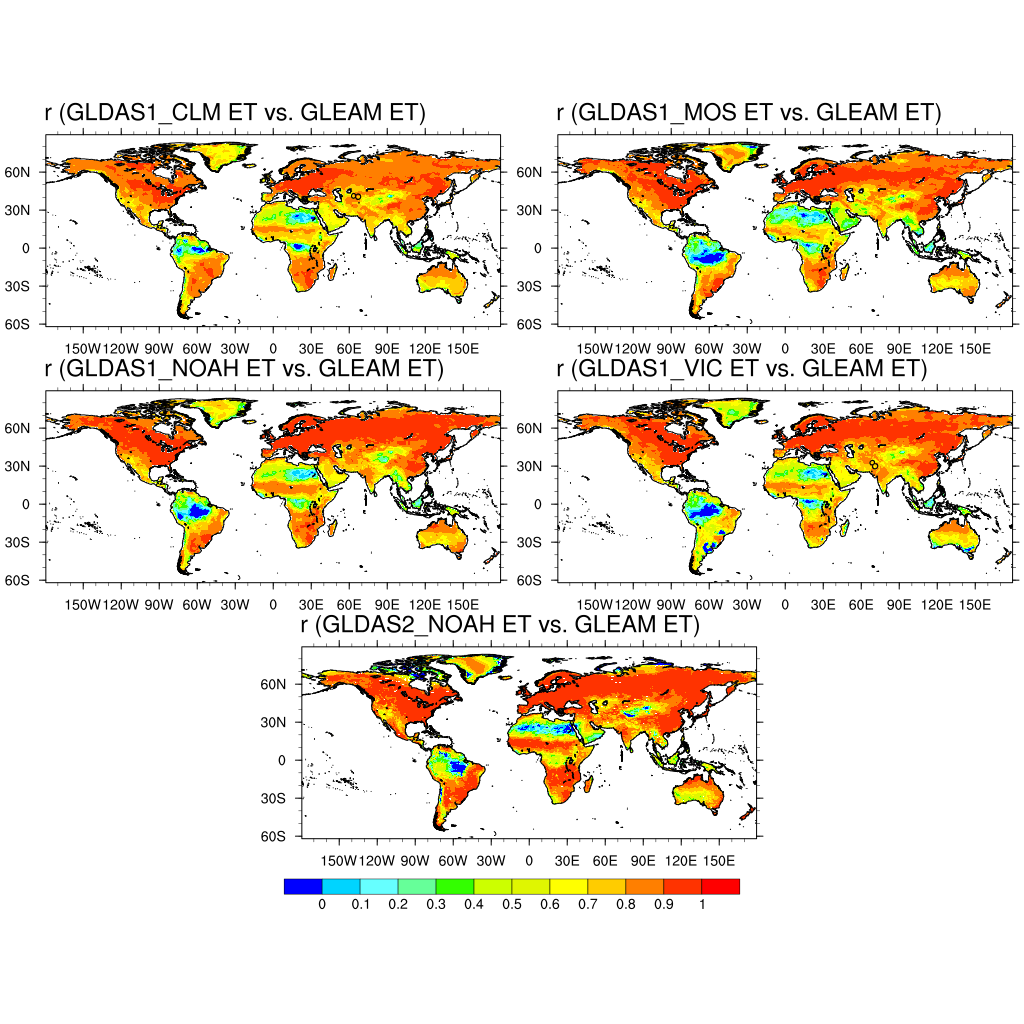


**Figure S3.** Global distribution of correlations between GLEAM *ET* and five GLDAS *ET* products for a 1980–2010 period.


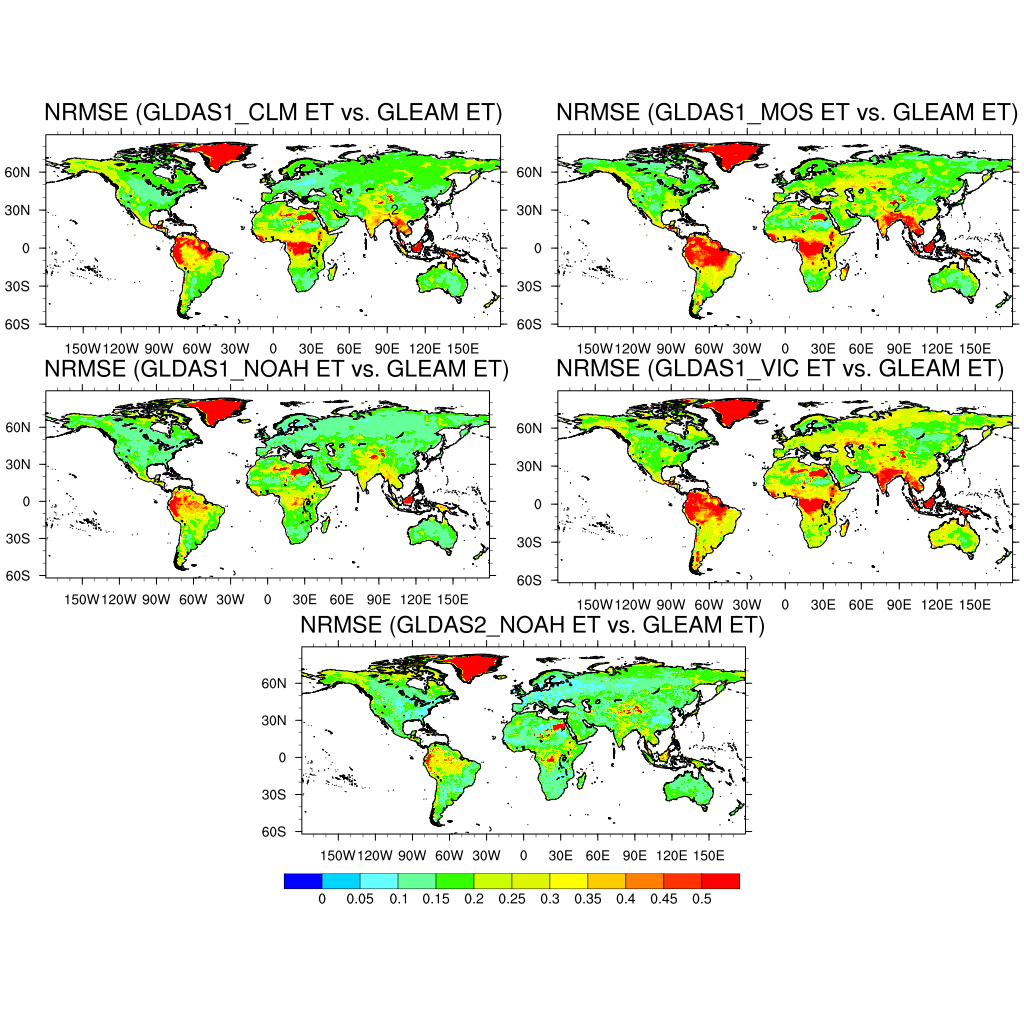


**Figure S4.** Global distribution of NRMSE between GLEAM *ET* and five GLDAS *ET* products for a 1980–2010 period.


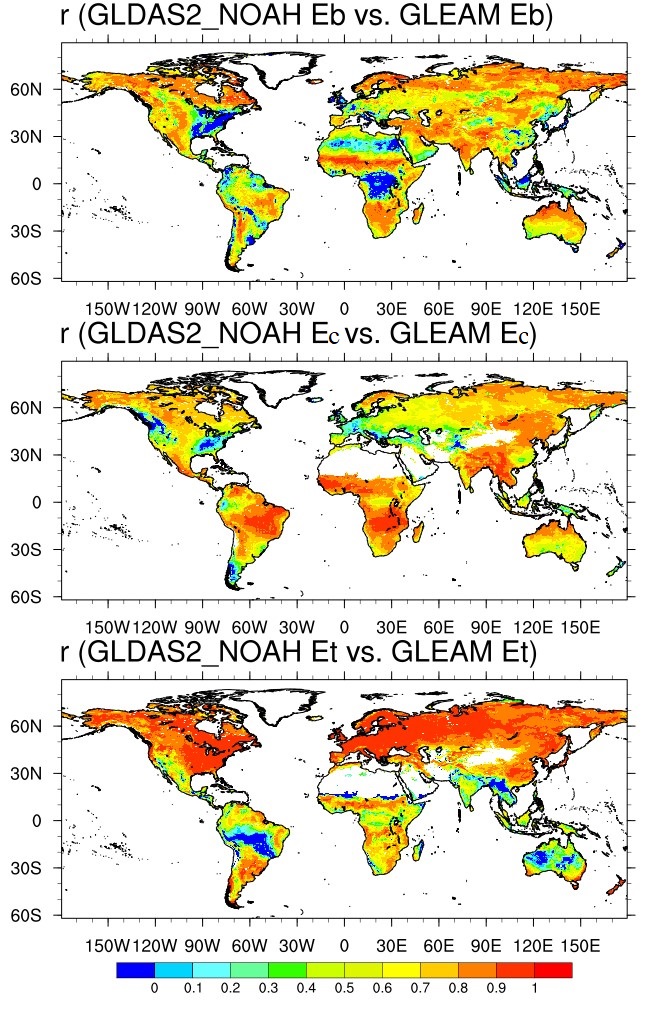


**Figure S5.** Global distribution of correlations between GLDAS2 NOAH and GLEAM for *E_b_*, *E_c_*, and *E_t_* over 1980–2010.


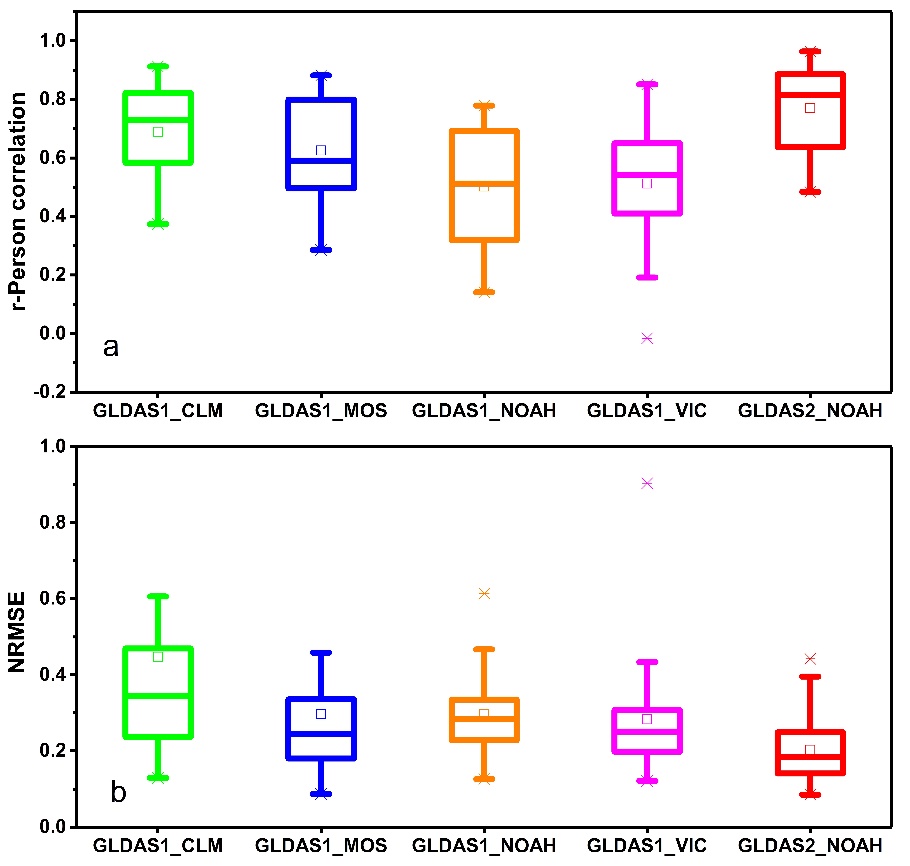


**Figure S6.** Boxplots showing the (a) correlations and (b) NRMSE between the GLDAS streamflow and the streamflow data from Pan et al. (2012) over 32 global large basins for a 1984–2006 period.


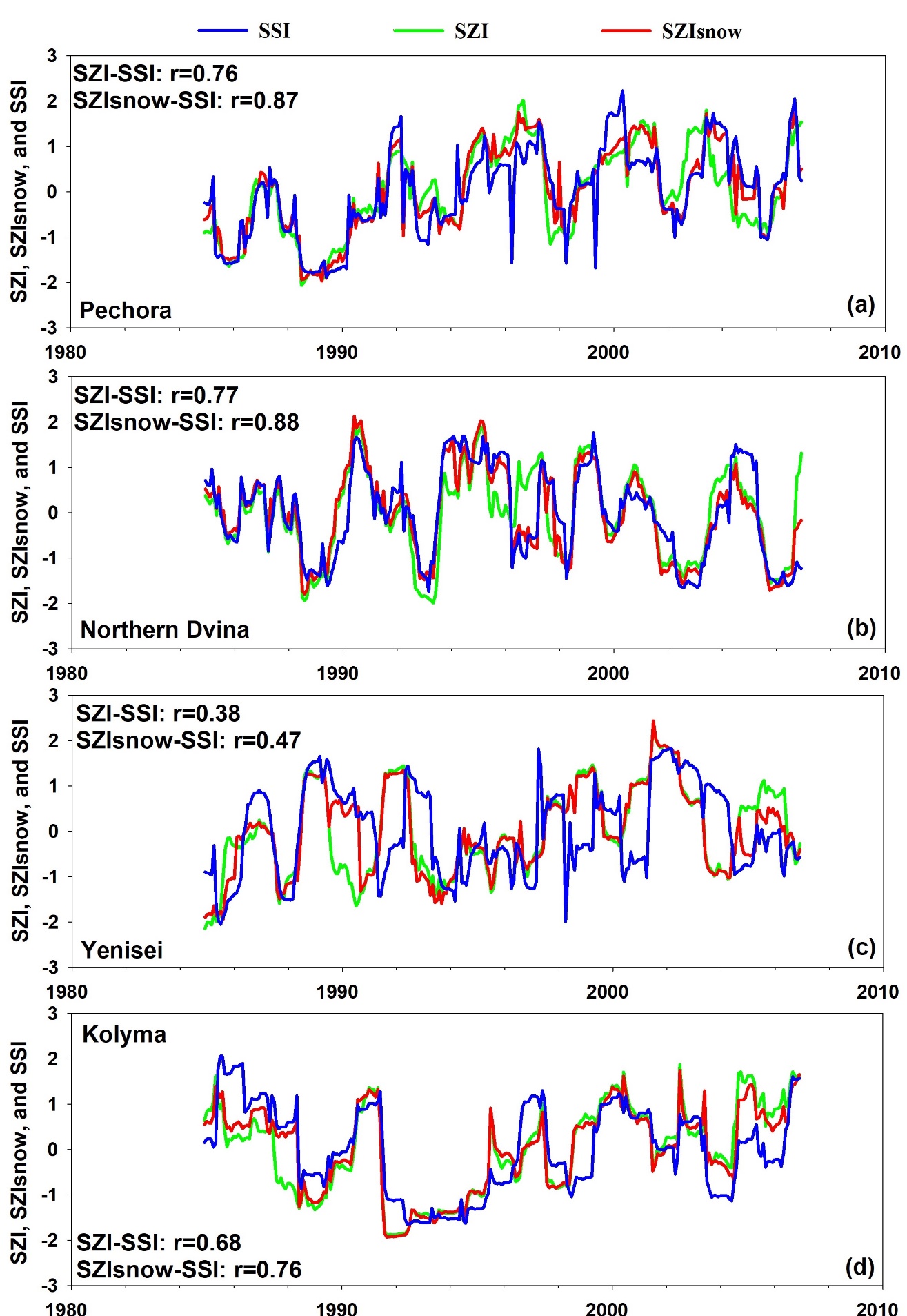


**Figure S7.** Comparison of the 12-month SSI, SZI, and SZI_snow_ time series at the Pechora, Northern Dvina, Yenisei, and Kolyma basins, respectively. The SPI, SZI, and SZI_snow_ are based on spatially-averaged input data over all grid cells in each basin.


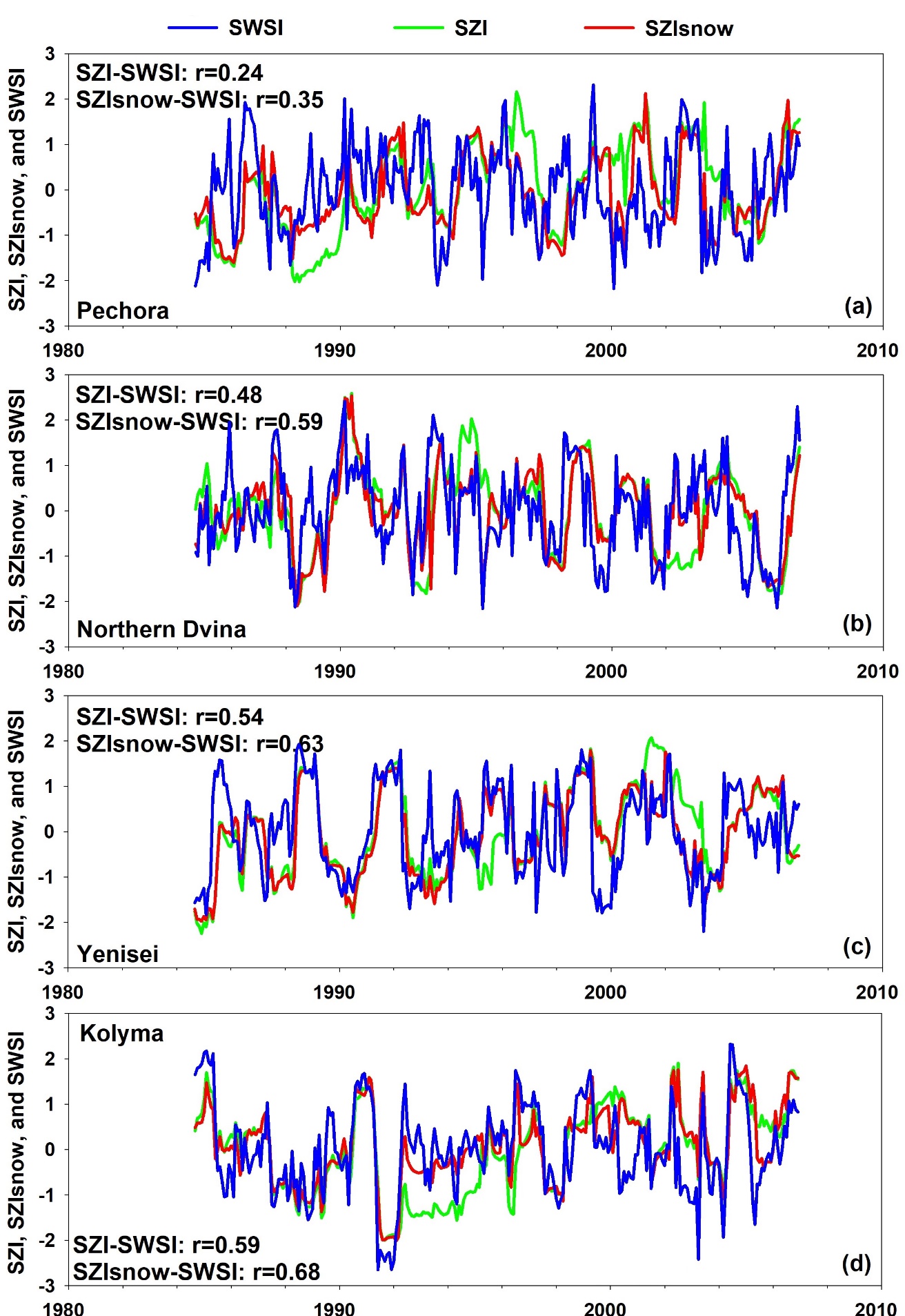


**Figure S8.** Comparison of the 9-month SWSI, SZI, and SZI_snow_ time series at the Pechora, Northern Dvina, Yenisei, and Kolyma basins, respectively. The SPI, SZI, and SZI_snow_ based on spatially-averaged input data over all grid cells in each basin.

**
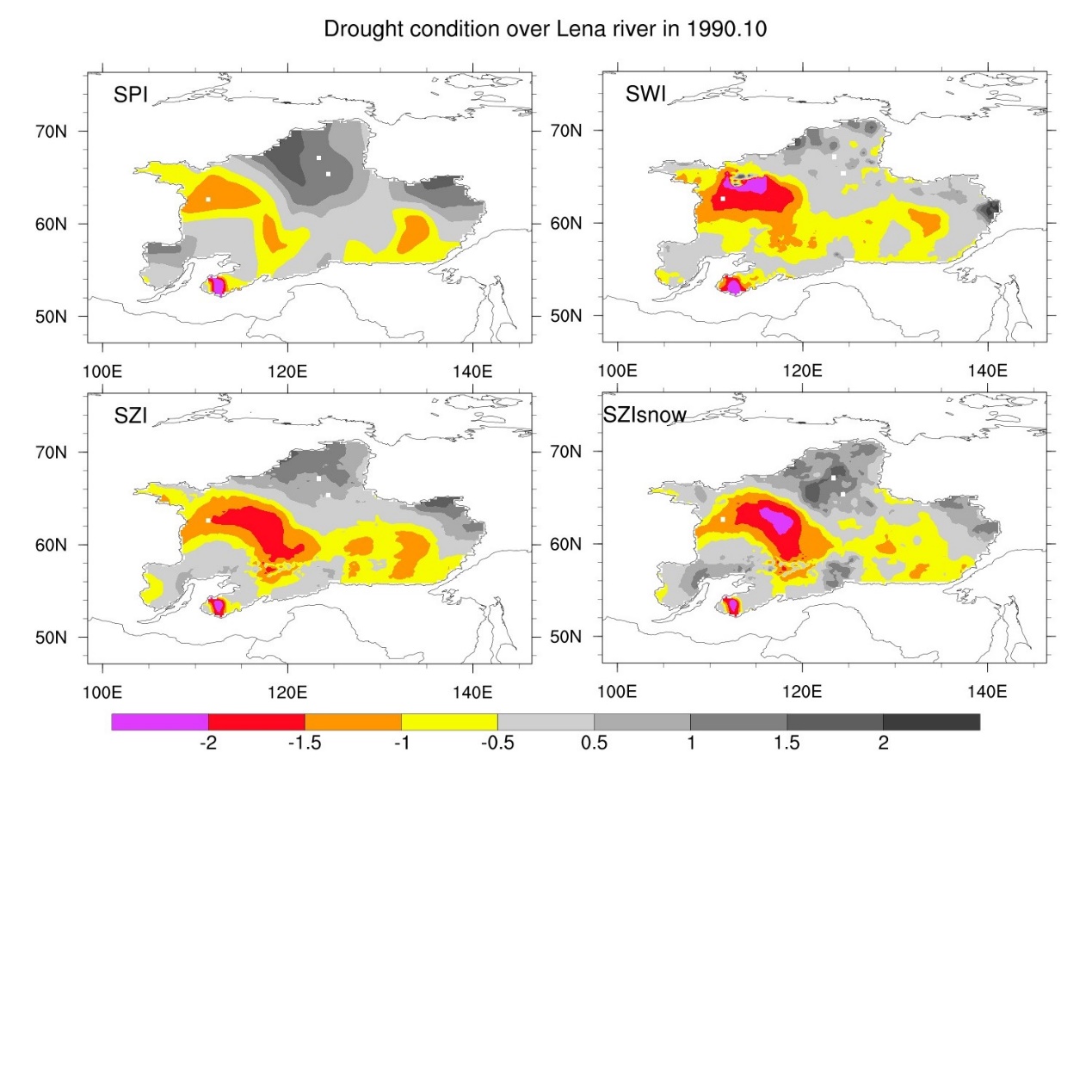
**

**
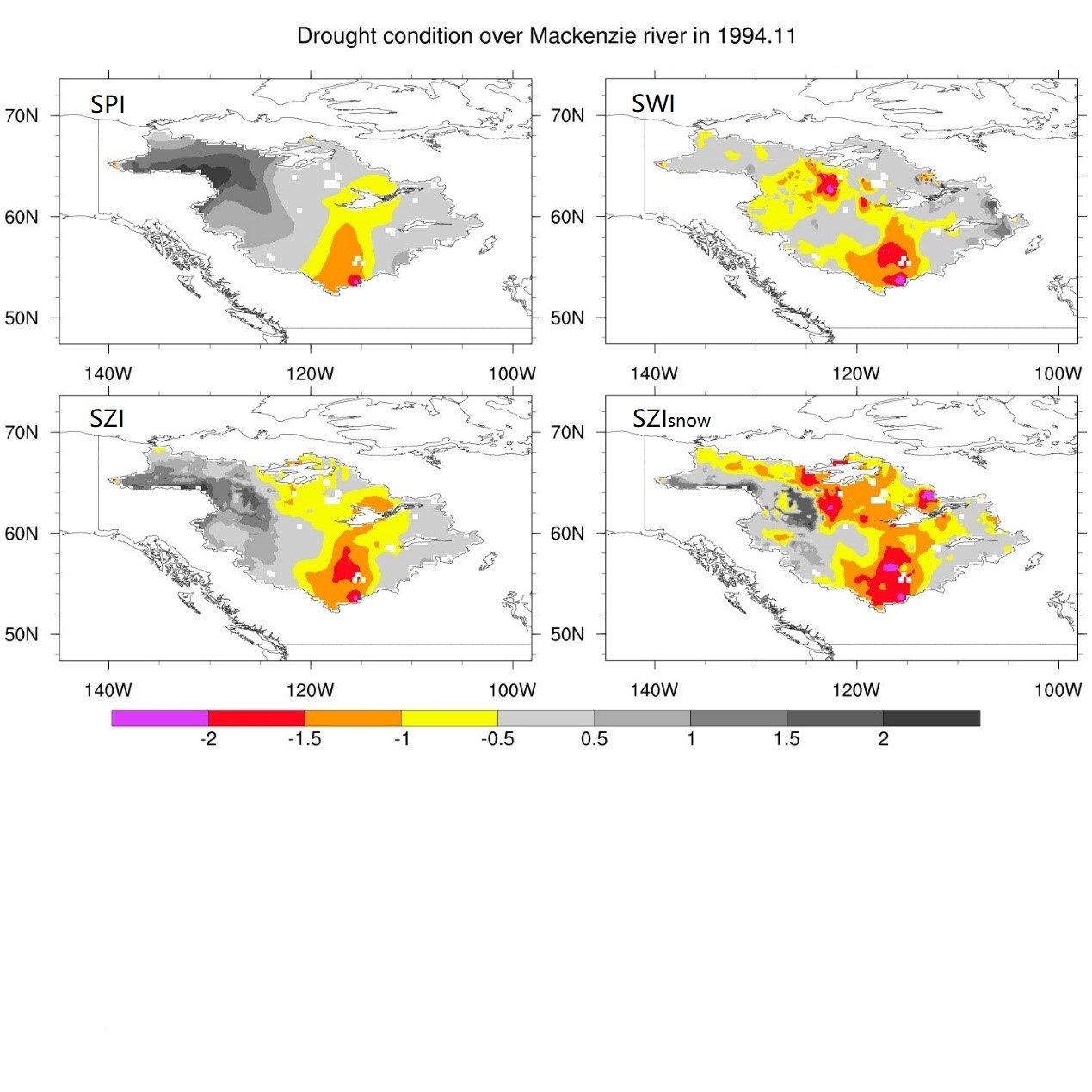
**

**
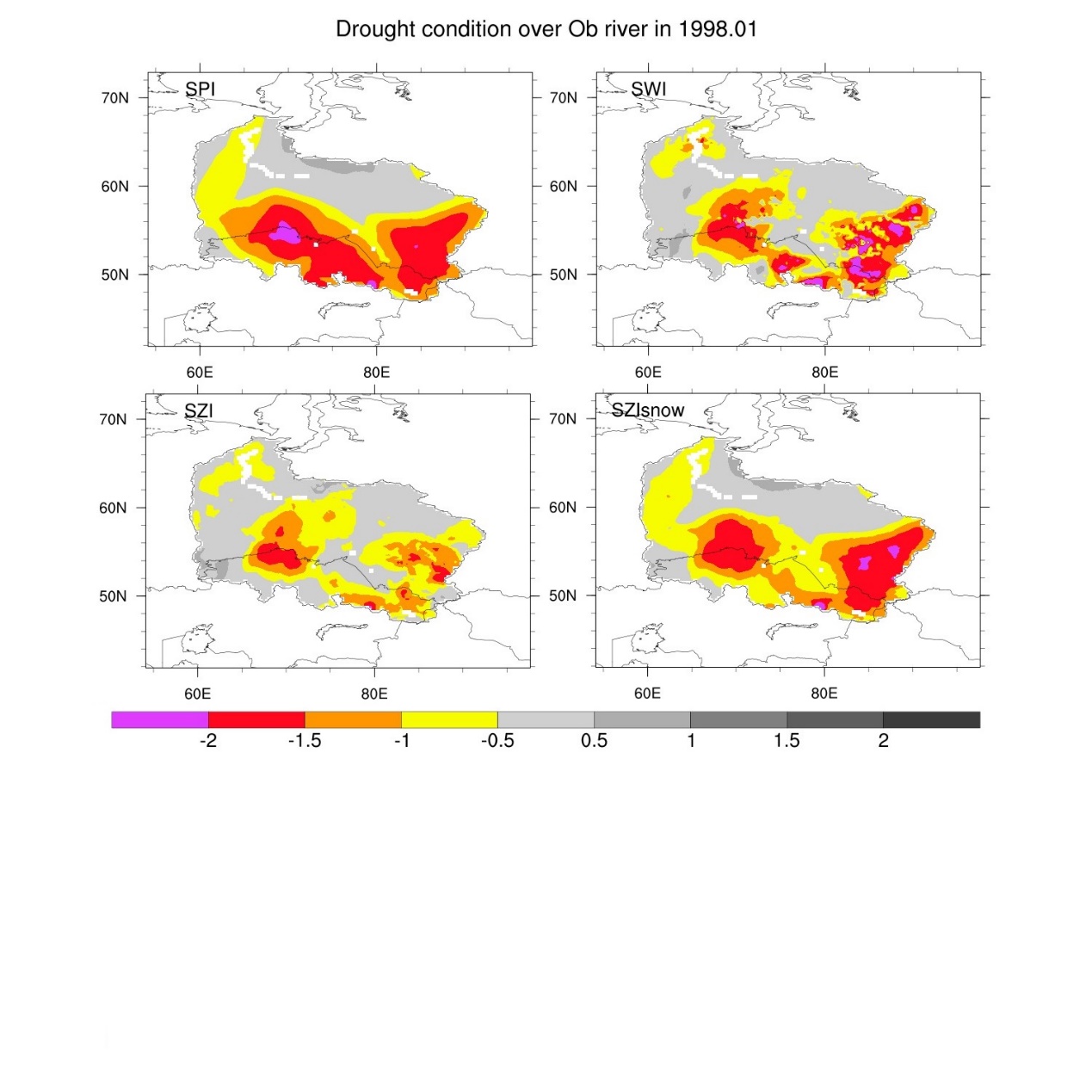
**

**
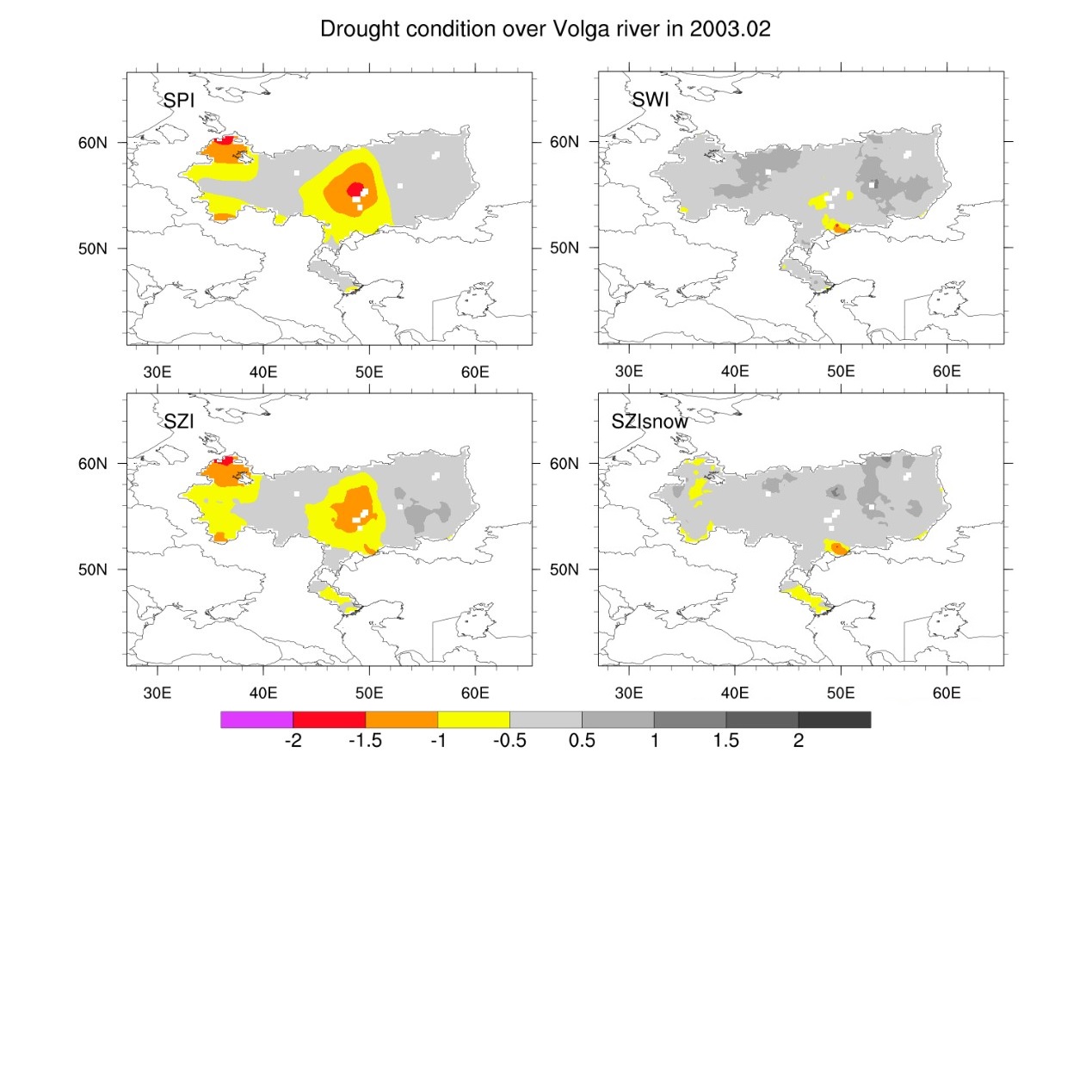
**

**
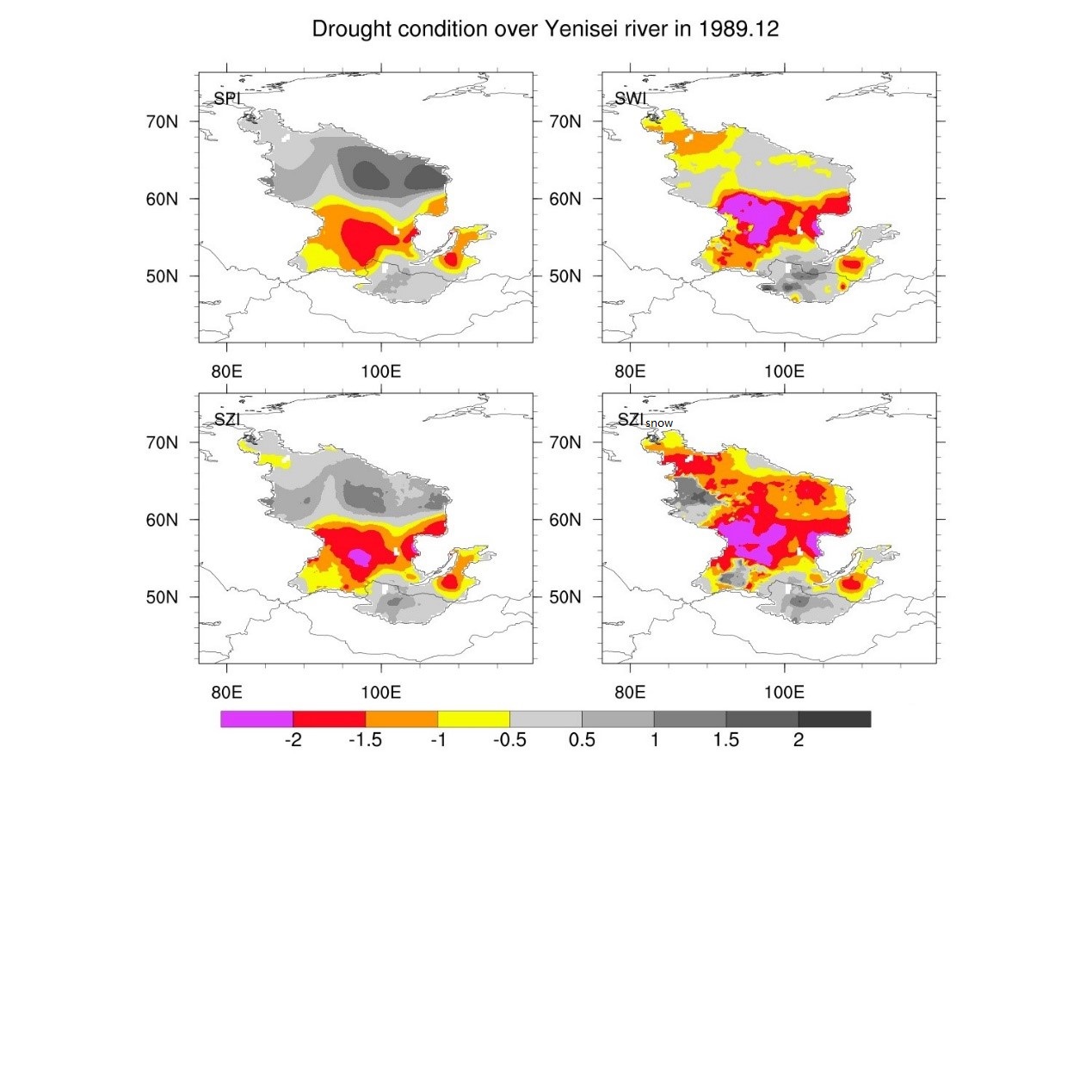
**

**Figure S9.** Comparison of identified droughts based on SPI, SWI, SZI, and SZI_snow_ in the Lena, Mackenzie, Ob, Volga, and Yenisei basins for select examples. All drought indices are at monthly scale, and the color bar represent the specific values of each drought index.
